# On the Mechanism of Chloroquine Resistance in *Plasmodium falciparum*

**DOI:** 10.1371/journal.pone.0014064

**Published:** 2010-11-19

**Authors:** Mauro Chinappi, Allegra Via, Paolo Marcatili, Anna Tramontano

**Affiliations:** 1 Department of Biochemical Sciences, Sapienza University, Rome, Italy; 2 Istituto Pasteur, Fondazione Cenci Bolognetti, Sapienza University, Rome, Italy; Dana-Farber Cancer Institute, United States of America

## Abstract

Resistance to chloroquine of malaria strains is known to be associated with a parasite protein named PfCRT, the mutated form of which is able to reduce chloroquine accumulation in the digestive vacuole of the pathogen. Whether the protein mediates extrusion of the drug acting as a channel or as a carrier and which is the protonation state of its chloroquine substrate is the subject of a scientific debate. We present here an analytical approach that explores which combination of hypotheses on the mechanism of transport and the protonation state of chloroquine are consistent with available equilibrium experimental data. We show that the available experimental data are not, by themselves, sufficient to conclude whether the protein acts as a channel or as a transporter, which explains the origin of their different interpretation by different authors. Interestingly, though, each of the two models is only consistent with a subset of hypotheses on the protonation state of the transported molecule. The combination of these results with a sequence and structure analysis of PfCRT, which strongly suggests that the molecule is a carrier, indicates that the transported species is either or both the mono and di-protonated forms of chloroquine. We believe that our results, besides shedding light on the mechanism of chloroquine resistance in *P. falciparum*, have implications for the development of novel therapies against resistant malaria strains and demonstrate the usefulness of an approach combining systems biology strategies with structural bioinformatics and experimental data.

## Introduction

In the last decades, due to its effectiveness and reasonable cost, chloroquine has represented the best and more widely used antimalarial drug. Unfortunately, within a decade of its introduction, *P. falciparum* parasite resistance to chloroquine was observed in most of the malaria-endemic countries. Nowadays, insurgence of resistance against chloroquine is a considerable hurdle for malaria control [1].

In its erythrocyte stage, *P. falciparum* invades the red blood cells where it forms a lysosomal isolated acidic compartment known as the digestive vacuole (DV). In the erythrocyte, the parasite grows by ingesting haemoglobin from the host cell cytosol and depositing it in the DV, where the protein is degraded to its component peptides and heme, which is incorporated into the inert and harmless crystalline polymer hemozoin [2].

Chloroquine is a diprotic weak base and, at physiological pH (∼7.4), can be found in its un-protonated (CQ), mono-protonated (CQ^+^) and di-protonated (CQ^++^) forms. The uncharged chloroquine is the only membrane permeable form of the molecule and it freely diffuses into the erythrocyte up to the DV. In this compartment, chloroquine molecules become protonated and, since membranes are not permeable to charged species, the drug accumulates into the acidic digestive vacuole [3], [4] where it is believed to bind haematin, a toxic byproduct of the haemoglobin proteolysis [5], [6], preventing its incorporation into the haemozoin crystal [2], [7], [8], [9], [10]. The free haematin seems to interfere with the parasite detoxification processes and thereby damage the plasmodium membranes [11].

Chloroquine sensitive parasites (CQS) accumulate much more chloroquine in the DV than chloroquine resistant strains (CQR) [4], [12], [13]. Recent studies have associated the reduced chloroquine accumulation observed in the parasite vacuole of resistant strains [12] with point mutations in the gene encoding for the *P. falciparum* chloroquine resistance transporter (PfCRT) protein (for a review see [14], [15]). PfCRT is localized in the digestive vacuole membrane and contains 10 predicted membrane-spanning domains [16], [17]. CQR phenotype isolates have all been found to carry the PfCRT critical charge-loss mutation K76T or, in two single cases, K76N or K76I [18], [19], [20], [21]. Another mutation, S163R, restores the chloroquine sensitivity of CQR parasites [22], [23]. The K76T amino acid mutation might allow the interaction of PfCRT with the positively charged chloroquine (CQ^+^ or CQ^++^) and allow its exit from the vacuole, with the net result of decreasing the chloroquine concentration within the DV [16], [24]. The single amino acid change S163R, by reintroducing a positive charge, is thought to block the leak of charged chloroquine from the DV, thus restoring chloroquine sensitivity [22], [23]. In a recent work, Martin and collaborators [25] were able to express both wild-type and resistant forms of PfCRT on the surface of *Xenopus laevis* oocytes and clearly demonstrated that chloroquine resistance is due to the direct transport of a protonated form of the drug out of the parasite vacuole via the K76T PfCRT mutant. Interestingly, they also showed that the introduction of the K76T single mutation in PfCRT of CQS parasites is necessary but not sufficient for the transport of chloroquine via PfCRT. These evidences are however compatible with two alternative models for PfCRT [26]: (1) the channel model (i.e. a passive channel that enables charged chloroquine to leak out of the food vacuole down its electrochemical gradient) or (2) the carrier model (i.e. an active efflux carrier extruding chloroquine from the food vacuole).

Several experimental set-ups have been used to answer the question of whether PfCRT is a channel or a carrier, namely measures of chloroquine accumulation, trans-stimulation and measures of chloroquine efflux. However the available data have been interpreted in different ways by different authors and the debate about the nature of PfCRT is still ongoing.

Sanchez and colleagues showed that chloroquine accumulation is energy dependent in both CQR and CQS [27]. These authors monitored the time course of labeled chloroquine uptake in the absence and in the presence of glucose. Glucose was added 20 min after choloroquine addition (i.e. when the stationary state was reached). They found that, after glucose addition, the time courses of choroquine uptake were markedly different in CQS and CQR: chloroquine accumulated to an increased extent in the CQS strain, but decreased in the CQR strain. A similar experiment was repeated by the same authors in 2004 [28] using a broader range of different antimalarial drugs. The authors concluded that the data are compatible with most models that attempt to account for chloroquine resistance and that some energy-dependent mechanism leads to loss of chloroquine from CQR cells and to its accumulation in CQS cells.

Bray et al [29], in 2006, measured the Cellular Accumulation Ratio (CAR) of chloroquine in six experimental conditions, namely in sensitive and resistant strains, in the absence and presence of carbonylcyanide p-trifluoromethoxyphenylhydrazone (FCCP), a ionophoric uncoupling agent, and in the absence and presence of glucose. In particular they found that, in absence of glucose, chloroquine the CAR is equal in CQS and CQR strains (∼700), reaching a level that is approximately intermediate between that observed in CQS (∼1200) and CQR (∼350) strains in the presence of glucose. They used several different Plasmodium strains and showed that, in the absence of FCCP, i.e. when the pH of the vacuole is lower than the external pH, the chloroquine CAR is three to four times higher (about 1200 versus about 350) in sensitive strains with respect to resistant strains, while addition of FCCP abolishes the differences leading to a CAR value of about 700 in both cases. They also demonstrate that, in the absence of glucose, the CAR is identical to that obtained in the presence of FCCP suggesting that the energy provided by the glucose is needed to maintain the pH difference between the cytoplasm and the DV. According to the authors, the hypothesis that PfCRT is an active efflux carrier does not appear to fully explain their findings. In this hypothesis, in fact, a single mutation would transform an energy-dependent chloroquine uptake process in an energy-dependent chloroquine efflux process. Therefore they favor the hypothesis that the chloroquine movement through PfCRT is not an active process.

Trans-stimulation of labeled chloroquine ([^3^H]-CQ)) uptake after the parasites were pre-loaded with increasing concentrations of unlabelled chloroquine [27], [28], [29], [30] was observed in CQR strains and not in CQS isolates. Sanchez and collaborators conclude that the trans-stimulation phenomenon is unequivocally characteristic of saturable, carrier-mediated transport systems [31]. On the other hand, Bray and colleagues [29] propose that the trans-stimulation data reported by Sanchez et al cannot by themselves be used to conclude whether the chloroquine transport is in the inward or outward direction: stimulation of [^3^H]-CQ uptake could indeed be due to acceleration of the transporter cycle by the outgoing unlabelled chloroquine or, as Sanchez et al assert, it could result from reduced efflux of [^3^H]-CQ due to the carrier competitive inhibition from the pre-loaded unlabelled CQ. In the latter model, labeled and unlabelled chloroquine should be on the same side of the membrane when they interact with the carrier, i.e. they are mixed together. In order to verify this hypothesis, Bray et al [29] incubated CQR lines with premixed chloroquine (labeled and unlabelled), but did not observe trans-stimulation of chloroquine uptake, thus suggesting that labeled and unlabelled chloroquine must be on opposite sides of the membrane for the trans-stimulation effect to take place, i.e. transport of unlabelled chloroquine via the carrier would be in the outward direction while labeled chloroquine transport would occur in the inward direction; in other words, mutant PfCRT would act as a bidirectional carrier, which is not compatible with an active efflux pump. In particular, these authors conjecture that trans-stimulation results might also be explained in terms of a gated channel.

Many authors measured chloroquine efflux from CQS and CQR isolates [27], [29], [32], [33] under different conditions: in presence and absence of glucose, with or without proton gradient uncoupling, with or without Verapamil, an L-type calcium channel blocker of the Phenylalkylamine class. The results of these experiments have been interpreted in different ways by different authors and did not lead to a consensus view about the nature of PfCRT.

Sanchez et al [34] studied the kinetics of chloroquine efflux in ‘reverse varying-trans’ conditions [31] from CQR and CQS isolates. This procedure investigates whether extracellular unlabelled chloroquine would stimulate the release of pre-loaded [^3^H]-CQ. These authors expected that, in the presence of an active carrier, trans chloroquine should increase the initial efflux rate. They found an increasing initial efflux rate for both CQR and CQS lines and accordingly proposed that both CQR and CQS parasites possess a carrier of chloroquine with different transport properties.

It should appear clear from this survey that qualitative interpretations of the experimental findings are insufficient to draw conclusions about the nature of PfCRT and that more quantitative analyses are required.

A quantitative model cannot be derived from transient experiments because kinetic parameters, such as the rate of the vacuole pH equilibration during chloroquine uptake and the kinetic constants of chloroquine-hemozoine binding, are unknown and impossible to extract from the available data in an unambiguous way. The only data that can be used to derive a quantitative model without making an unreasonable number of hypotheses on the unknown parameters are those measured at equilibrium.

As we will show, the analytical model that we developed and used here indicates that equilibrium data are compatible with both the carrier and channel model for PfCRT, which explains why they could be interpreted differently by different authors. On the other hand, the carrier and channel hypotheses are only compatible with specific assumptions on the protonation state of the transported species and of the species binding to haeme of haeme-related molecules in the vacuole. For example, a carrier model is only compatible with the data if the transported molecule is protonated.

Another route to understand the nature of the macromolecule is to study its evolutionary relationship with other proteins of known function. Also in this case, data interpretation is controversial. Previous computational analyses of PfCRT [14], [16] suggested that PfCRT belongs to the drug/metabolite trasporter (DMT) superfamily, whereas other studies proposed that it resembles to ClC chloride channels [35].

Here, we use state-of-the-art bioinformatics tools to identify PfCRT homology relationships and provide evidence that it is indeed a member of the DMT superfamily This finding is also supported by the observation that a three-dimensional model of the protein based on a DMT-like fold is consistent with experimental data about the mutations involved in insurgence and reversion of CQ resistance in Plasmodium.

By combining this latter conclusion with the results of the analytical method, we propose that PfCRT is a carrier of CQ^+^, CQ^++^ or both and that either all chloroquine species or only the uncharged one can bind hame of hame related species inside the vacuole.

## Results

We selected to use the data from the Cellular Accumulation Ratio (CAR) experiments described by [29] because, as mentioned above, the experimental conditions (stable and controlled pH values) allow the model to be built using a number of parameters comparable with the number of observations.

Our approach consists in testing the consistency of all plausible hypotheses about the binding mode of the drug to the heme related species inside the vacuole and the mechanism of action of PfCRT with experimental data. Our reasoning does not require any assumption on which heme form or heme related molecule binds to chloroquine and, for this reason, we refer to the heme related species bound by chloroquine inside the vacuole as HM.

Two mechanisms are thought to be involved in chloroquine accumulation into the *P. falciparum* vacuole: acidic trapping due to low vacuolar pH and chloroquine binding to heme or heme related species. It is reasonable to assume that PfCRT does not directly affect the molecular mechanism of chloroquine-HM binding. Recent studies [36], [37], [38] indicate that the vacuolar pH of CQS and CQR strains are similar, hence the reduction of chloroquine accumulation in resistant strains cannot be explained in terms of different acidic trapping and PfCRT must be directly involved in releasing chloroquine out of the vacuole. A proof of this hypothesis has been recently provided by Martin and collaborators [25].

### The analytical approach

In this study we formulate an analytical model describing different combinations of the two sets of hypotheses described below, the first related to the mode of chloroquine binding to HM, the second to the mechanism of action of the mutated PfCRT. We test all possible combinations of these hypotheses for their consistency with the available experimental data provided by [29] and summarized in Table 1. Abbreviations used throughout the manuscript are reported in Table 2.

**Table 1:** Values of the chloroquine cellular accumulation ratio in sensitive and resistant strains, in the presence and absence of FCCP, as estimated from figures 1A and 2A in Bray et al [29].

CAR	−FCCP	+FCCP
Sensitive strain (CQS) GC03 strain	∼1200 (B)	∼700 (A)
Resistant strain (CQR) Dd2 strain	∼350 (C)	∼700 (D)

**Table 2:** List of abbreviations used throughout the paper.

Abbreviation	Description
CQS	Cloroquine Sensitive Strain
CQR	Cloroquine Resistant Strain
CAR	Cellular Accumulation Ratio
CQ	un-protonated form of chloroquine
CQ^+^	mono-protonated form of chloroquine
CQ^++^	di-protonated form of chloroquine
CQ^TOT^	CQ+CQ^+^+CQ^++^
CQ*	it is used in equations that hold for all four forms of chloroquine (CQ, CQ^+^, CQ^++^ and CQ^TOT^)
[^3^H]-CQ	labeled chloroquine
DV	Digestive Vacuole
HM	heme related species bound by chloroquine inside the vacuole
[CQ], [CQ^+^], [CQ^++^], [CQ^TOT^]	Concentration of CQ, CQ^+^, CQ^++^, CQ^TOT^
[CQ∶HM]_DV_	concentration of the complex chloroquine:HM inside the vacuole
[H^+^]	concentration of H^+^
FCCP	carbonylcyanide
p-trifluoromethoxyphenylhydrazone, a ionophoric uncoupling agent	
J_PfCRT_	chloroquine flux through PfCRT
HMM	Hidden Markov Model

Concerning the chloroquine binding to HM we consider two possibilities:

#### H1) The concentration of the complex chloroquine:HM inside the vacuole, [CQ∶HM]_DV_, linearly increases with the concentration of the binding form of chloroquine in the vacuole

According to which chloroquine species reacts with HM, this can be expressed as [CQ∶HM]_DV_ = α [CQ]_DV_ or [CQ∶HM]_DV_ = α [CQ^+^]_DV_, or [CQ∶HM]_DV_ = α [CQ^++^]_DV_ or [CQ∶HM]_DV_ = α [CQ^TOT^]_DV_, where [CQ^TOT^] = [CQ]+[CQ^+^]+[CQ^++^]. The latter representing the case of chloroquine binding HM regardless of its protonation state.

#### H2) The concentration of the complex chloroquine:HM increases non linearly with the concentration of the binding chloroquine form in the vacuole and reaches saturation at concentrations of chloroquine above a given threshold

If we define the threshold concentrations as t_HM,CQ_, t_HM,CQ+_, t_HM,CQ++_ or t_HM,CQtot_ or simply t_HM_ when the species is clear from the context, the above hypothesis can be expressed as: [CQ∶HM]_DV_ = f([CQ]_DV_) that, for CQ_DV_>t_HM,CQ_, reads [CQ∶HM]_DV_ = constant. Similar expressions hold for [CQ∶HM]_DV_ = f([CQ^+^]_DV_), [CQ∶HM]_DV_ = f([CQ^++^]_DV_) and [CQ∶HM]_DV_ = f([CQ^TOT^]_DV_).

As far as PfCRT is concerned, the two possible cases are:

#### J1) PfCRT acts as a passive channel for CQ^+^ (or CQ^++^, or both)

and thereby the outward flux of chloroquine across the vacuole membrane due to PfCRT, J_PfCRT_, only depends upon the difference in concentration on the two sides of the vacuolar membrane and on the membrane potential. For instance, if the membrane potential is zero and the channel allows CQ^+^ to move out of the DV, J_PfCRT_ = f([CQ^+^]_DV_−[CQ^+^]_e_), the suffix “e” indicating the plasmodium cytoplasm. As shown in the Text S1, section S1, being the vacuole membrane freely permeable to un-protonated chloroquine CQ, the hypothesis that PfCRT acts as a channel for CQ alone can be immediately discarded since it is not consistent with the experimentally observed differences between CQR and CQS.

#### J2) PfCRT is a carrier for CQ, (or CQ^+^, or CQ^++^)

In this case, it is reasonable to assume that the flux through the channel is a linear function of the CQ (or CQ^+^, or CQ^++^) concentration for concentration values below a given threshold and a constant above the threshold concentration. Let us define the threshold concentrations as t_PfCRT,CQ_, t_PfCRT,CQ+_, or t_PfCRT,CQ++_ or simply t_PfCRT_ when the species is clear from the context. The above hypotheses can be expressed as:
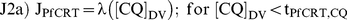


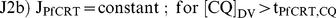
similar expressions hold for J_PfCRT_ = f([CQ^+^]_DV_), J_PfCRT_ = f([CQ^++^])_DV_.

The procedure adopted to test all combinations of the above hypotheses for their consistency with the available experimental data makes use of the analytical expressions for the membrane equilibrium, for the Cellular Accumulation Ratio (CAR) of chloroquine, which is the quantity measured in the experiments described in [29] and for the basis dissociation equilibrium.

The membrane equilibrium equation is:
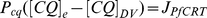
(1)where P_cq_ is the membrane permeability to unprotonated chloroquine; equation (1) has been obtained taking into account that the system has reached a steady state in the analysed experimental conditions. Consequently, the net chloroquine flux across the membranes is zero. The only form of chloroquine for which the erythrocyte and the external plasmodium membranes are permeable is the un-protonated one and this implies that CQ concentrations are the same on the two sides of these membranes.

The expression for CAR is:
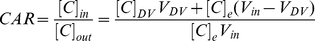
(2)where the total concentration of chloroquine inside the vacuole is:
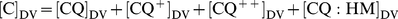
(3)being [CQ∶HM]_DV_ the concentration of chloroquine bound to HM.
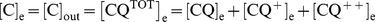
(4)is the concentration of chloroquine outside the vacuole, [C]_in_ is the average chloroquine concentration in the infected erythrocyte, V_in_ is the volume of the infected erythrocyte and V_DV_ is the volume of the digestive vacuole. The analytical derivation of eqs (1) and (2) is detailed in Text S1, sections S2 and S3, respectively. The proof of the first equality of eq (4) ([C]_e_ = [C]_out_) is reported in Text S1, section S4.

Finally, the relationships between the concentrations of the three forms of chloroquine given by the two-base dissociation equilibrium are:
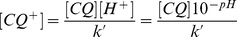
(5)

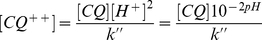
(6)with 1/k′ = (1/k_2_+1/k_2_) and k″ = k_1_k_2_ and k_1_ and k_2_ are the two dissociation constants.

Experimentally determined values for the constants used in Eqs (2) to (6) are reported in Table 3.

**Table 3:** Values of the parameters used throughout the paper.

Parameter	Description	Value	Reference
C_out_ = C_e_	external chloroquine concentration	2 nM	[29]
k_1_	chloroquine dissociation constants	10^−8.1^	[53]
k_2_	chloroquine dissociation constants	10^−10.2^	[53]
V_in_	volume of the erythrocyte	80 fL	[10]
V_DV_	volume of the vacuole	4 fL	[10]
P_cq_	vacuole membrane permeability	7.5 cm/s	[54] [55]
pH_e_	external (physiological) pH	7.4	[29]
pH_DV_	vacuole pH	4.5–4.95.18±0.055.4–5.5	[36] [37] [38]

**Figure 1:**
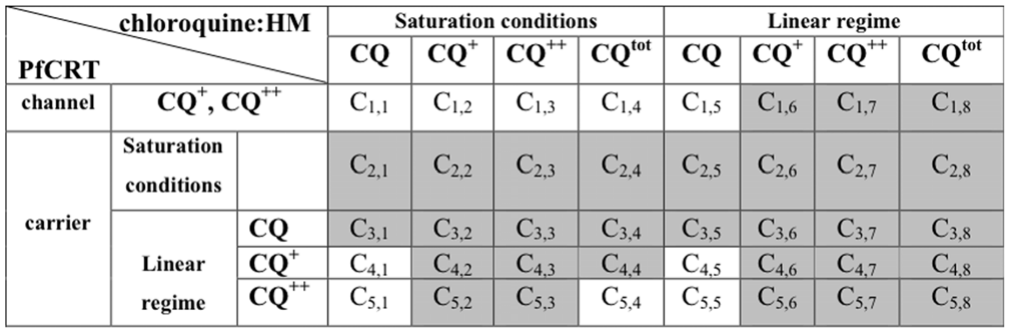
Summary of the hypotheses analysed throughout the manuscript. The columns refer to the mode of binding of the chloroquine to HM, the rows to mutated PfCRT, considering the possibility that the latter is a channel or an active carrier. Shaded cells correspond to combination of hypotheses inconsistent with the analysed data.

Figure 1 summarizes the various hypotheses tested here. The columns refer to the mode of binding of the chloroquine to HM, assuming either that the experimental data have been obtained in non saturation conditions (linear regime, hypothesis H1) or that the concentration of the chloroquine species is above the (unknown) saturation concentration for HM (saturation conditions, hypothesis H2). The rows are related to assumptions about the mutated PfCRT function considering the possibility that the latter is a channel (first row, hypothesis J1) or an active carrier (remaining rows, hypotheses J2) and, in the case of active carrier, considering the possibility that the considered chloroquine species concentration into the vacuole is or is not above the (unknown) threshold needed to saturate PfCRT.

In the following we will show the procedure used to test the hypotheses summarized in Figure 1. We use the symbol CQ* in equations that hold for all four forms of chloroquine (CQ, CQ^+^, CQ^++^ and CQ^TOT^).

**Figure 2:**
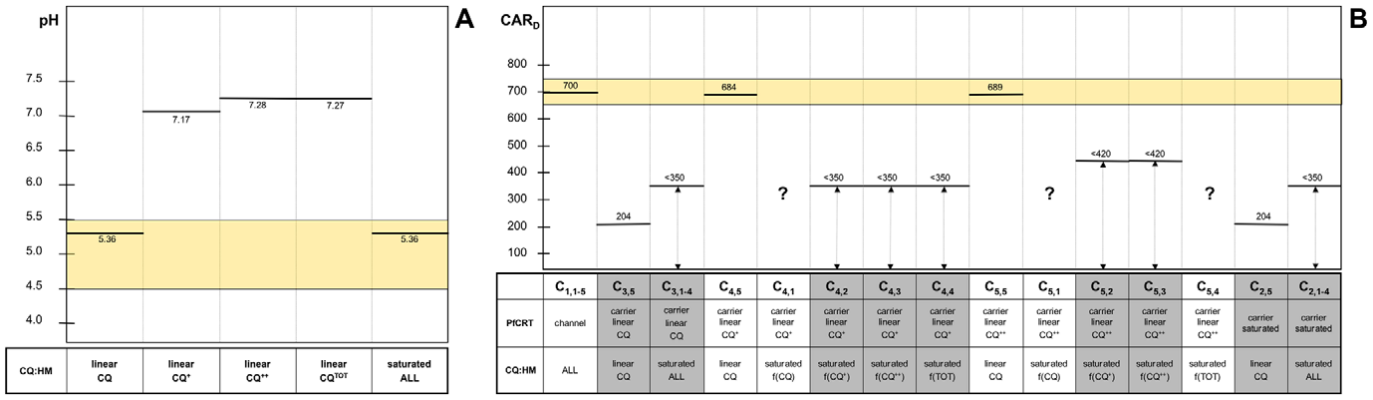
Graphical representation of the results of the analytical model in comparison with the experimental data. A) Computed values for the pH of the vacuole in the hypothesis that the concentration of the complex of HM with the indicated CQ species linearly increases with the concentration of the ligand (first four columns) or that the system is at saturation (last column). ALL refers to either CQ, CQ^+^, CQ^++^, CQ^TOT^. Orange shaded area indicates the range of experimental values and their uncertainty measured by different authors in different experiments. As described in the text, the calculation only uses the CAR for sensitive strains (experiments A and B in Table 1) and therefore does not require any hypothesis on the nature of the mutated PfCRT. B) Computed values of the CAR compared with observed values obtained in experiment D (see Table 1). The different hypotheses are shown in the table at the bottom. The first row refers to the cells of the table in Figure 1. The second row reports the tested hypotheses on the mechanism of PfCRT (channel or carrier), on whether the binding of PfCRT with the indicated species is in the linear regime (linear) or at saturation (saturated) and on the protonation state of the transported molecule (CQ: neutral; CQ^+^: mono-protonated; CQ^++^: di-protonated). The third row refers to the tested hypotheses for the binding of the listed chloroquine species (CQ, CQ^+^, CQ^++^ or CQ^TOT^; ALL refers to either CQ, CQ^+^, CQ^++^, CQ^TOT^.) with HM in the hypotheses that the latter complex is at saturation or in the linear regime. Grey shaded cells indicate that the computed values are incompatible with the experimental ones. The orange shaded area indicates the range of experimental values and their experimental error. Dashed arrows indicate that the model provides an upper limit for the value. Question marks indicate that no conclusion can be derived using the indicated combination of hypotheses.

We examined the consequences of each of the hypotheses reported in Figure 1. As a general strategy, we used the values of CAR in experiments A, B and C reported in Table 1 (CAR_A_, CAR_B_ and CAR_C_, respectively) to calibrate the model, inferred a CAR value for experiment D (CAR_D_) and compared it with the experimental one. In some cases, the value of CAR_A_ and CAR_B_ was used to infer the value of the vacuolar pH (pH_DV_) subsequently compared with the experimental one. When the results show that a hypothesis is inconsistent with the experimental data and has to be discarded, the corresponding cell in Figure 1 is shaded. Inferred values of CAR_D_ and their agreement or disagreement with experimental values are shown in Figure 2B.

#### Test of hypothesis H1 ([CQ∶HM]_DV_ = α [CQ*]_DV_)

Here, we use the values of CAR_A_ and CAR_B_ to infer the pH_DV_ value. Notice that, since CAR_A_ and CAR_B_ refer to CQS strains, here we are not making any assumption on PfCRT.

Let us consider the hypothesis that the concentration of the complex CQ*∶HM linearly increases with the vacuolar concentration of CQ*, i.e. hypothesis H1 corresponding to the twenty cells C_1–5,5–8_ in Figure 1 in the columns labeled as “Linear regime”. We can have four different expressions for the total chloroquine accumulated in the vacuole [C]_DV_ depending on which chloroquine form reacts with HM, namely
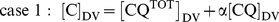
(7)

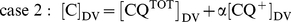
(8)

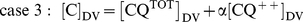
(9)

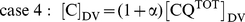
(10)corresponding to columns 5, 6, 7 and 8 of Figure 1, respectively.

Combining these equations with (2), (3), (4), (5), and (6) an expression for CAR as a function of the binding constant α and of pH_DV_ can be obtained. For instance, in case 1 (eq. (7)), we have:
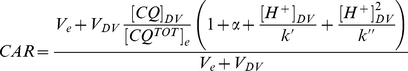
(11)where V_e_ = V_in_−V_DV_.

Let us consider the behavior of CQS strains. In this case, [CQ]_DV_ = [CQ]_e_ ((eq-S3) Text S1, section S2) and, in the presence of FCCP, [H^+^]_DV_ is equal to [H^+^]_e_. Hence, by substituting the known value of CAR for sensitive strains (experiment A in Table 1), we can compute the parameter α. Knowing α and using the experimental value of CAR in the absence of FCCP (experiment B in Table 1) we can solve the second order equation (11) in pH_DV_, which is found to have only one positive solution. The same procedure can be applied to equations (8–10) to derive the equivalent of (11) for cases 2–4 (see also Text S1, section S5). The results for the four cases are:
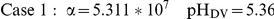


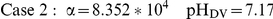


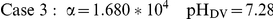


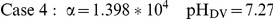



Only the pH_DV_ value obtained in case 1 is compatible with the experimental data for pH_DV_ (Figure 2A and Table 3) and therefore we can conclude that, if the data are obtained in non saturating conditions for HM binding, the non protonated form of chloroquine binds to HM, regardless of the mechanism of action of the mutated PfCRT. The C_1–5,6–8_ cells in Figure 1 are therefore shaded since they correspond to cases not compatible with the experimental data.

#### Test of hypotheses J1 (PfCRT is a channel) and J2 (PfCRT is a carrier) [cells C_1–5,5_ Figure 1 and Figure 2B]

Let us now consider the data obtained for resistant strains (C and D in Table 1). We can use the values of pH_DV_ and α to calculate the concentration of the un-protonated chloroquine inside the vacuole for CQR strains in the absence of FCCP (experiment C Table 1) using equation (11). We call this value [CQ]_DV,C_ to indicate that it is related to experiment C and use eq (1) to calculate the outward flux due to PfCRT, i.e.
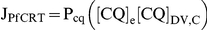
obtaining 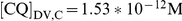 and 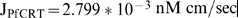.

Next, we examine the specific hypotheses for PfCRT.

If PfCRT is a channel and the DV membrane potential is zero, the transport is only driven by the difference in concentration of the transported chloroquine form.

As a paradigm of our procedure, we describe the case where the transported form is CQ^+^. A similar reasoning can be applied if the transported species is CQ^++^ or a combination of CQ^+^ and CQ^++^.

We have
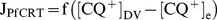
(12)The function f(x) is increasing and it is zero when its argument is zero. The presence of FCCP in experiment D (Table 1) implies that pH_DV_ = pH_e_, i.e. that the the concentrations of the three chloroquine forms are the same inside and outside the vacuole. Combining this observation with eq. (12) and the membrane balance equation (eq. (1)), we obtain that [CQ]_DV,D_ = [CQ]_e_, [CQ^+^]_DV,D_ = [CQ^+^]_e_ and [CQ^++^]_DV,D_ = [CQ^++^]_e_, i.e. both the chloroquine diffusive flux through the vacuolar membrane and through PfCRT (eq (12)) are zero (for detailed calculations see Text S1, section S6). These findings imply that CAR_D_ = CAR_A_, which is consistent with the experimental data (Figure 2B). Accordingly, cell C_1,5_ of Figure 1 and Figure 2B is not shaded.

Notice that, as a by-product of our calculations, the J_PfCRT_ permeability can be explicitly calculated in the hypothesis that f(x) is a linear function of ([CQ^+^]_DV_−[CQ^+^]_e_) (see Text S1, section S7).

The argument used to demonstrate that CAR_D_ = CAR_A_ when the membrane potential is zero also holds if the membrane potential is different from zero. According to [26], [29] we assume that the DV membrane potential is mainly due to a proton gradient; therefore, the presence of the proton un-coupler FCCP in experiments A and D (Table 1) lowers the membrane potential to a negligible value. On the other hand, a non zero membrane potential should only be taken into account when analyzing the results of experiments B and C, whose data are not used here to demonstrate that CAR_D_ = CAR_A_. In other words, the presence of a non zero membrane potential, that would require the addition of a term in equation (12), does not affect experiments A and D. Consequently it would have no effect on eq (12) and on our whole reasoning.

For testing the hypothesis that PfCRT is a carrier, corresponding to cells C_2–5,5_ in Figure 1, we need to consider the case of the CQ* concentration inside the vacuole being lower (or not) than the threshold value t_PfCRT_, i.e. being insufficient (or sufficient) to saturate the carrier (hypotheses J2a and J2b).

In the first case, corresponding to cells C_3–5,5_ in Figure 1, the outward flux is a linear function of the concentration of the transported chloroquine form CQ*, i.e.
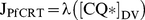
(13)As shown in Text S1, section S8, the CAR_D_ values for each of the chloroquine forms can be computed from eqs. (1), (11) and (13). In particular:
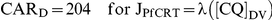


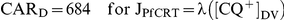


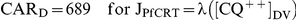
Corresponding to cells C_3–5,5_ in Figure 1, respectively. The only cases where the results are consistent with the data reported in Table 1 are that PfCRT is an active carrier for either CQ^+^ or CQ^++^ in a linear regime (Figure 2B). This implies that only cell C_3,5_ is shaded in Figure 1 and Figure 2B.

A similar line of reasoning allows the CAR_D_ value to be computed in the hypothesis that the CQ* concentration inside the vacuole is not lower than the threshold value t_PfCRT_. In this case (corresponding to cell C_2,5_ in Figure 1) we have
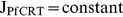
(14)In particular, J_PfCRT,C_ = J_PfCRT.D_ where the suffix C and D refer to the experiments shown in Table 1. Eq. (14) and the CQR membrane balance equation (eq (1)) implies [CQ]_DV,C_ = [CQ]_DV,D_. By substituting the values for [CQ]_DV,D_ and α in equation (11) and remembering that pH_DV_ = pH_e_ in the presence of FCCP, we obtain CAR_D_ = 204. This value significantly differs from the experimental one indicating that hypothesis J2b is not compatible with the data (Figure 2B). Accordingly, the corresponding cell C_2,5_ in Figure 1 and Figure 2B is shaded.

#### Test of the hypothesis H2 ([CQ∶HM]_DV_ = constant)

We first use the values of CAR_A_ and CAR_B_ to infer the pH_DV_ value and verify its consistency with the experimentally known one. Notice that, since CAR_A_ and CAR_B_ refer to CQS strains, we are not initially making assumptions on PfCRT.

Let us test the possibility that [CQ*] is above the concentration t_HM,CQ_ needed to saturate the HM binding sites in the experiment with sensitive strains in the presence of FCCP (Experiment A in Table 1). As a first step, we need to understand whether this is the case also for the experiment performed on sensitive strains in the absence of FCCP (Experiment B in Table 1).

Because of the presence of FCCP in experiment A, we have:
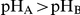
and, consequently, [CQ]_DV,A_ = [CQ]_DV,B_, [CQ^+^]_DV,A_<[CQ^+^]_DV,B_ and [CQ^++^]_DV,A_<[CQ^++^]_DV,B_, which implies that, in B, the concentration of CQ* is either the same as in A or higher and, therefore, [CQ∶HM]_DV,B_ must be in the saturation regime, where [CQ∶HM]_DV_ = constant, as well. It follows that:
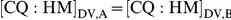
Similarly to the linear regime hypothesis for HM binding (hypothesis H1), it is possible to use equations (2), (3), and (4) to rewrite the expression for CAR as follows:
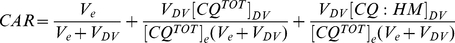
(15)where [CQ^TOT^]_x_ (with *x* being either *DV* or *e*) is the sum of the three free chloroquine forms, namely [CQ^TOT^]*_x_* = [CQ]*_x_*+[CQ^+^]*_x_*+[CQ^++^]*_x_* = [CQ]*_x_* (1+[H^+^]*_x_*/k′+[H^+^]^2^ *_x_*/k″). Note that equation (15) is a general expression for CAR that, in the hypothesis H1, reduces to (11). The value of [CQ∶HM]_DV,A_ can now be calculated using the experimental value of CAR_A_, while pH_DV,B_ can be obtained from equation (15) (where the dependence on pH_DV_ is embedded in the [CQ^TOT^]_DV_ term). The only positive solution admitted by the equation is pH_DV,B_ = 5.36, which is consistent with the experimental data (Figure 2B and Table 3). This finding, obtained without making any assumption on the nature of PfCRT, implies that hypothesis H2 is plausible, regardless of the chloroquine species involved in the chloroquine:HM binding and of the mechanism of action of the mutated PfCRT. Consequently, none of the 20 cells C_1–5,1–4_ can be shaded at this stage.

#### Test of hypotheses J1 (PfCRT is a channel) and J2 (PfCRT is a carrier) [cells C_1–5,1–4_ Figure 1]

As shown before, if experiment A has been performed in saturation conditions, the same is true for experiment B. We now need to infer what are the conditions of experiment C, i.e. whether
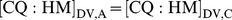
(16)Notice that in experiment A there is no effect of PfCRT (being this the case of a sensitive strain) or of pH differences (because of the presence of FCCP), therefore all the chloroquine accumulation is due to the chloroquine bound to HM. In particular, it is apparent from eq. (15) that, without the [CQ∶HM]_DV_ contribution, CAR_A_ would be 1. Equation (16) implies that the [CQ∶HM]_DV_ contribution to CAR_C_ is equal to the [CQ∶HM]_DV_ contribution to CAR_A_ hence, due to the additional contribution of pH to chloroquine accumulation in C, we would have CAR_C_>CAR_A_. Since the experimental data show that CAR_C_<CAR_A_, we can reject the hypothesis represented by equation (16) and conclude that the concentration of chloroquine in experiment C is not in the saturation region (detailed numeric calculation can be found in Text S1, section S9). In conclusion, if experiment A was performed at saturating chloroquine:HM concentration, the same holds for experiment B, but not for experiment C. This implies that, in experiment C, we have [CQ∶HM]_DV_ = f(CQ*), where f(CQ*) is a monotonic increasing function of one of the chloroquine species.

In the hypothesis that PfCRT is a channel, the reasoning is identical to that of section 1.1.1 for cell C_1,5_ of Figure 1 and leads to the same conclusions: we cannot exclude that PfCRT is a channel (Figure 2B) therefore, cells C_1,1–4_ in Figure 1 are not shaded. Notice that no hypothesis on the chloroquine:HM binding is required in the reasoning of section 1.1.1

We now discuss the case when the transported form is CQ^+^, i.e. PfCRT is an active carrier that transports CQ^+^ in the linear regime (cell C_4,1–4_ Figure 1). A similar reasoning can be applied if the transported species are CQ or CQ^++^; the detailed calculations for these other cases are reported in Text S1 (sections S11 and S12).

In the hypothesis that PfCRT transports CQ^+^ and that the dependency of the J_PfCRT_ flux is linear in the concentration of vacuolar CQ^+^ (J_PfCRT_ = λ[CQ^+^]_DV_) we need to consider the following cases for chloroquine-HM binding:
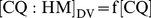
(17a)

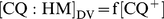
(17b)

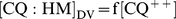
(17c)

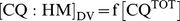
(17d)corresponding to cells C_4,1–4_ of Figure 1, respectively.

Equation (15) clearly shows that the difference between CAR_C_ and CAR_D_ is due to its second and third terms. Combining the expression for the PfCRT flux J_PfCRT_ = λ([CQ^+^]_DV_) with that of the membrane equilibrium (eq. (1)) we have that [CQ]_DV_ = [CQ]_e_/(1+[H^+^]_DV_ λ/(k′ P_cq_) ), and hence
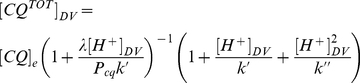
(18)that is, for any value of the unknown parameter λ, an increasing function of [H^+^]_DV_ in the interval 0<pH_DV_<9.15, which is the interesting one for plasmodium metabolism (the proof is reported in Text S1, section S10). Being [H^+^]_DV,C_>[H^+^]_DV,D_, we have [CQ^TOT^]_DV,C_>[CQ^TOT^]_DV,D_; therefore the second member of equation (15) is larger for case C than for case D. As far as the third member of the equation is concerned, we have to distinguish the four cases (17a–d). Detailed calculations relating to each single hypothesis for the chloroquine:HM binding (cases (17a–d)) are reported in Text S1, section S13. Taking into account that [H^+^]_DV,C_>[H^+^]_DV,D_ and that f(CQ*) is monotonic, we eventually obtain (Figure 2B):
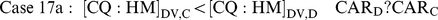


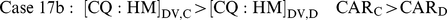


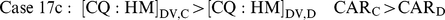


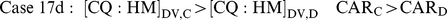
While in the first case no conclusion can be drawn on the relationship between CAR_C_ and CAR_D_ (indicated by a question mark in Figure 2B), cases (17b), (17c) and (17d) can be excluded and the corresponding cells C_4,2_, C_4,3_ and C_4,4_ can be shaded in Figure 1 and Figure 2B.

If J_PfCRT_ = *constant* (cells *C_2,1–4_* in Figure 1), this expression can be used in the vacuolar membrane balance equation for CQR strains (eq. (1)). Following the reasoning reported in Text S1, section S14, we obtain that CAR_D_<CAR_C_, which is inconsistent with the experimental data (Figure 2B). This implies that hypothesis H2 in conjunction with the hypothesis that PfCRT is a saturated chloroquine carrier is not plausible. Accordingly, cells C_2,1–4_ (Figure 1 and Figure 2B) are shaded.

We tested how stable our model is with respect to reasonable variations of the CAR values. We repeated the whole procedure using values of CAR derived for other parasite lines and for variation within the experimental error obtaining the same conclusions (data not shown).

In conclusion, ten of the forty cells of Figure 1, each corresponding to a different assumption about the mode of binding of chloroquine to HM in conjunction with a specific form of PfCRT, are not shaded, i.e. they correspond in principle to hypotheses consistent with the experimental data.

### Sequence and structure analysis of PfCRT

Previous bioinformatics analyses of the PfCRT protein were devoted at identifying the functional role of the PfCRT protein, leading to different conclusions. On one hand several authors [14], [16] assigned PfCRT to the drug/metabolite transporter (DMT) superfamily and, among the previously defined protein families, reported that PfCRT has the highest similarity with the drug/metabolite exporter (DME) family. On this basis, the authors concluded that PfCRT is likely to function as an exporter of metabolites in symport with H^+^.

Other studies proposed that PfCRT might share significant sequence similarity with the ClC chloride channels of other organisms [29], [35], which would reinforce the hypothesis that the protein acts as a gated aqueous pore.

As already mentioned by other authors [14], data supporting the ClC similarity hypothesis are not available and were impossible to reproduce using any available sensitive and updated sequence analysis tool. On the contrary, as detailed later, several different and effective methods strongly support the hypothesis that PfCRT is an active carrier.

**Figure 3:**
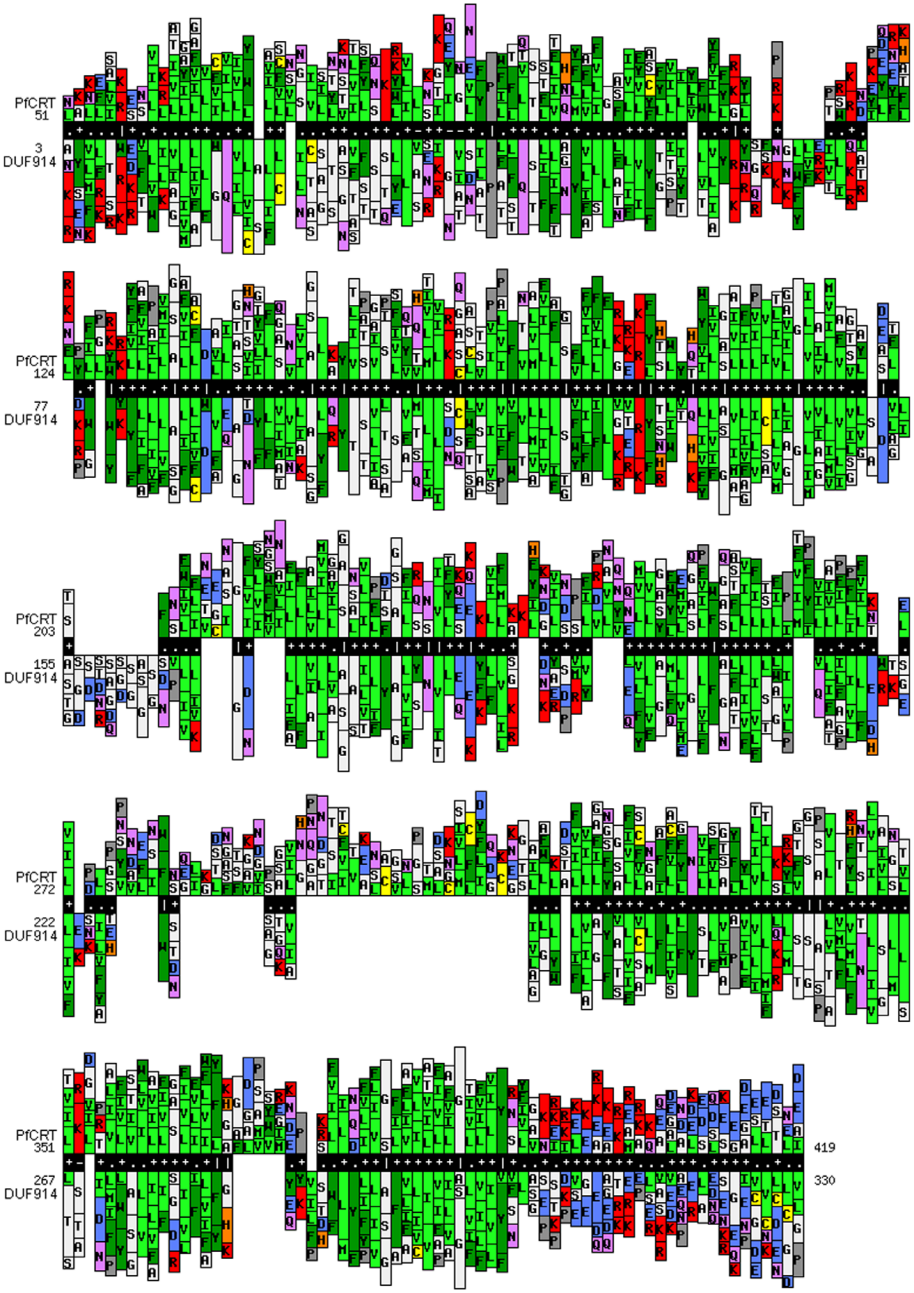
Alignment between the PfCRT family and the DUF914 family. Profile-profile alignment of PfCRT homologs (see Materials and Methods) and the Pfam family DUF914, a member of the DMY clan. Residues are colored according to their physico-chemical properties (green = hydrophobic, red = negatively charged, blue = positively charged, pink = polar, white = small, yellow = cysteine, gray = proline, orange = histidine). The image was generated using the MPI Bioinformatics Toolkit [52].

The HHsearch tool [39], [40] identifies three protein families in the Pfam database [41] with the highest similarity to PfCRT: PF06027 (DUF914, E-value 1.2×10^−34^), PF08449 (*UAA* transporters, E-value 5.6×10^−24^), PF04142 (nucleotide-sugar transporters, E-value 7.8×10^−23^). These all belong to the Drug/Metabolite Transporter clan CL0184, which consists of several families of different secondary carriers, among which drug, sugar and nucleotide antiporters have the highest similarity with PfCRT. The alignment of PfCRT homologous with the closest Pfam family (DUF914) is shown in Figure 3.

Two amino acids of PfCRT are known to be involved in chloroquine resistance, 163 and 76, and therefore expected to be involved in the transport mechanism. To verify whether this is indeed the case, we built a homology model of the protein.

By searching the PDB database [42] with HHsearch, we retrieved two significant hits (e-value<0.1). The significant matches correspond to two solved structures (pdb codes 3B5D and 2I68) of the same protein, EmrE. This protein is a small *E.coli* multidrug transporter belonging to the same drug/metabolite transporter family described above that acts by exchanging various positively charged aromatic drugs across the plasma membrane with protons. EmrE is a homodimer, each monomer being composed by four membrane spanning helices. Two different regions of PfCRT sequence match three helices of each EmrE monomer, consistently with the presence of an internal sequence symmetry in PfCRT. We also searched for homologous proteins spanning the complete sequence of the protein to use as templates. Three different and sensitive methods (Shrimp [43], Phyre [44] and PROCAIN [45]) all identified the Glycerol-3-phosphate transporter GlpT from E.Coli (PDB code 1PW4) as a homolog of known structure with a significant sequence similarity spanning the whole sequence (e-value<10^−4^ in all cases). The protein was ranked first in the searches performed with Shrimp and PROCAIN and second by Phyre. The top ranking protein identified by Phyre (LacY) has the same fold and belongs to the same superfamily as the Glycerol-3-phosphate transporter.

GlpT was therefore selected as the template for the model. It is an active transporter presenting a 12-helix trans-membrane helix protein with an internal sequence symmetry.

**Figure 4:**
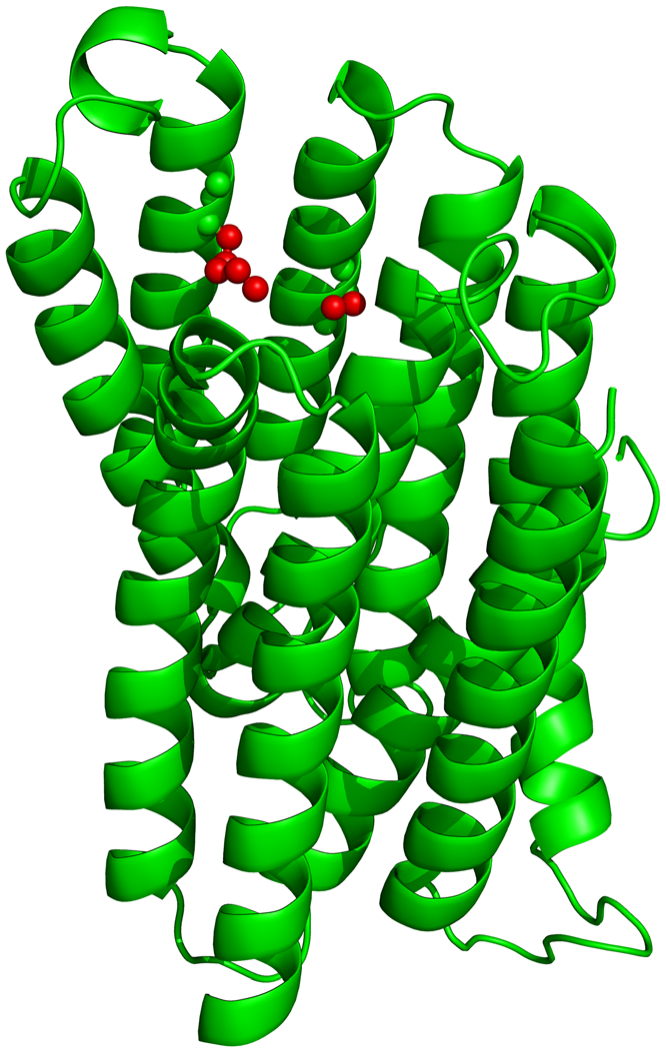
The model of the transmembrane region of PfCRT. Residues K76 (left side) and S163 (right side) are shown in red using a stick representation.

**Figure 5:**
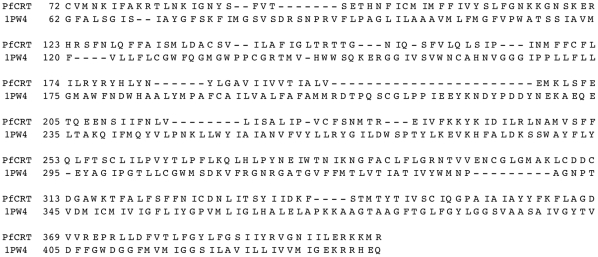
Sequence alignment between the target PfCRT protein sequence and the sequence of the template (1PW4) as obtained by Phyre.

The final model (shown in Figure 4) was generated according to the target/template alignment shown in Figure 5. The model coordinates can be downloaded from the PMDB database [46] (Id PM0076214).

Mapping of residues 163 and 76 in the three-dimensional model of the protein (Figure 4) shows that they face each other and line the path of the transported molecule. This is consistent with the experimental observations and it appears plausible that the K76T and S163R mutations can permit positive charged species to be transported or not, respectively.

In conclusion, sequence analysis strongly suggests that PfCRT is an active carrier belonging to the Drug/Metabolite transporter superfamily and, furthermore, a three-dimensional model based on the basis of this evolutionary relationship is consistent with the experimental data on the protein.

## Discussion

The ongoing debate on PfCRT, the molecule responsible for chloroquine resistance in Plasmodium, has not yet provided a conclusive answer to the question of whether PfCRT is a channel or a carrier.

The qualitative analysis of chloroquine accumulation, trans-stimulation and chloroquine efflux data has been used to support both the channel and the carrier hypotheses by different authors [26], [27], [29], [30], [34]. This is not surprising since we can analytically demonstrate that, at least as far as the choloroquine accumulation ratio experiments are concerned, the data are consistent with both hypotheses. A similarly rigorous approach cannot be used for the other experiment types since the problem is underdetermined.

This notwithstanding, we are able to show here that, if PfCRT is a carrier, it can only transport protonated or di-protonated chloroquine molecules and that choloroquine can either bind heme or heme related species in the digestive vacuole regardless of its charge or in its neutral form.

On the other hand, a reassessment of the evolutionary relationships of the protein with state-of-the-art methods and updated databases strongly suggests that the protein is indeed a member of the Drug/Metabolite transporter clan. This conclusion is further substantiated by the observation that a model based on this detected evolutionary relationship is consistent with experimental data.

Taking together the results of these two interdisciplinary approaches allow us to conclude that the chloroquine species are transported out of the vacuole through PfCRT in their protonated form, in agreement with studies such those presented by Lehane et al [24], [47] who provided evidence that the presence of chloroquine increases the leak of H+ from the vacuole.

We would like to emphasize that our analytical approach does not require *ad-hoc* hypotheses as it would be the case if we were to model data coming from trans-stimulation and chloroquine efflux experiments where parameters such as the rate of the vacuole pH equilibration during chloroquine uptake or the kinetic constants of chloroquine-hemozoine binding are unknown. On the other hand, the model can be used effectively to interpret the results of stationary experiments. As an example, recently Martin and collaborators [25] set up a system in which PfCRT is expressed at the surface of *Xenopus leavis* oocytes and measured the chloroquine uptake which, in this system, is not influenced by chloroquine-HM binding. The available data have been obtained in the pre-stationary state, but the same system could in principle be used to measure chloroquine uptake at equilibrium in a pH-controlled experiment. In this case a simplified version of our analytical model could be employed to derive the systems parameters and provide more detailed information about PfCRT.

Remarkably, our analysis conclusively demonstrates that experimental data on the chloroquine accumulation ratio at equilibrium are consistent with both hypotheses that the mutant molecule is an active or a passive carrier of the drug and therefore insufficient to distinguish between the two mechanisms, while at the same time they can be used to restrict hypotheses on the nature of the transported and HM-binding species in the two cases.

Our computational analysis of the sequence and a structural bioinformatics approach strongly suggests that the mutated protein acts as an active carrier of chloroquine and, in this assumption, we can conclude that the PfCRT mutated protein confers resistance by carrying either the mono or di-protonated chloroquine out of the vacuole. It is tempting to speculate that the mechanism is that the mutated PfCRT uses the H+ gradient to expel the protonated form of chloroquine from the vacuole. Cationic transport inhibitors could be tested to further support this hypothesis and, perhaps, as starting point for developing novel therapies against resistant malaria strains.

We hope that the example of the power of systems and computational biology analysis of the data presented here will convince the Plasmodium community to take advantage of our results.

## Materials and Methods

### Ethics Statement: N/A

We performed sequence similarity searches on the nr database [48] release of October 4^th^, 2010 using three iterations of CS-Blast [49]. We selected the first 500 hits, all having an e-value lower than 1e-5, and realigned their sequences using the multiple sequence alignment tool MUSCLE [50]. Such alignment was then used to scan the Pfam [41] and PDB [42] databases on October 5^th^, 2010 with the Hidden Markov Model based search method HHpred [39], the results of which are reported in Table S1. Three distant homology recognition tools, Phyre [51], Shrimp [43] and PROCAIN [45], were used to identify templates spanning the complete PfCRT sequence (see Table S2, Table S3 and S4). In all the cases, a statistically significant similarity of the PfCRT sequence with that of the Glycerol-3-Phosphate transporter GlpT from E.Coli (PDB code 1PW4) spanning the whole sequence of both target and template proteins was detected.

## Supporting Information

Text S1 Details of the calculations presented in the main manuscript.
(1.24 MB PDF)

Table S1 HHpred results on Pfam and PDB databases.
(0.02 MB PDF)

Table S2 Phyre results.
(0.02 MB PDF)

Table S3 PROCAIN results.
(0.02 MB PDF)

Table S4 Shrimp results.
(0.03 MB PDF)
