# Supplementary material for: On the Mechanism of Chloroquine Resistance in Plasmodium falciparum
Source: PLoS One. 2010 Nov 19;5(11):e14064. doi: 10.1371/journal.pone.0014064 (PMC2988812; doi:10.1371/journal.pone.0014064)
Supplement: Text S1 — Details of the calculations presented in the main manuscript. (1.24 MB PDF) [file pone.0014064.s001.pdf]

On the mechanism of chloroquine resistance in *Plasmodium falciparum*

Supporting Information

Mauro Chinappi[1*], Allegra Via[1*], Paolo Marcatili[1] and Anna Tramontano[1,2,+]

[1]Department of Biochemical Sciences, Sapienza University, Rome, Italy

[2]Istituto Pasteur, Fondazione Cenci Bolognetti, Sapienza University, Rome, Italy

Table of Contents

S1 - PfCRT cannot be a channel for un-protonated chloroquine CQ
S2 - Membrane equilibrium equation
S3 - The expression for the Cellular Accumulation Ratio of chloroquine
S4 - Chloroquine concentration is equal in the external buffer and inside the erythrocyte
S5 - CAR expressions in the case of chloroquine-HM binding in linear regime (hypothesis H1)
S6 - Proof that $[CQ]_{DV,D} = [CQ]_e$ in the hypothesis PfCRT is a channel for the protonated forms of chloroquine
S7 - Value of the channel permeability in the hypothesis PfCRT is a channel and $[CQ:HM]_{DV} = \alpha[CQ]_{DV}$
S8 - Calculation of CAR_D in the hypothesis $[CQ:HM]_{DV} = \alpha[CQ]_{DV}$ (H1) and PfCRT is a non saturated active carrier (J2a) (main text, Figure 1, cells C3-5,5)
S9 - Detailed numerical calculation showing that chloroquine-HM binding in experiment C is not in the saturation region
S10 - Proof that eq. (18) (main text) is an increasing function of $[H^+]_{DV}$ in the interval $0 < pH_{DV} < 9.15$
S12 - Calculation of CAR_D in saturated condition for chloroquine-HM binding and $J_{PfCRT} = \lambda[CQ^{++}]_{DV}$ (cells C5,1-4, Figure 1 of the main text])
S13 - Calculations relating to each single hypothesis for the chloroquine:HM binding in saturated condition and $J_{PfCRT} = \lambda\ [CQ^+]_{DV}$ (cells C4,1-4, Figure 1 of the main text])
S14 - Prediction for CAR_D in saturated condition for chloroquine-HM binding (H2) and J_{PfCRT} = const (J2b) (cells C2,1-4,Figure 1, main text)

S1 - PfCRT cannot be a channel for un-protonated chloroquine CQ

At equilibrium, the net flux of chloroquine across the vacuolar membrane is zero. Let J_{mem} be the inward membrane flux of unprotonated chloroquine CQ and J_{PfCRT} the chloroquine outward flux due to the CQR mutated form of PfCRT. We have:

$$J_{mem} = J_{PfCRT} \qquad \text{(eq-S1)}$$

[*] These authors contributed equally

In the hypothesis that PfCRT is a passive channel for CQ, both J_{mem} and J_{PfCRT} are only functions of the difference of CQ concentrations on the two sides of the vacuolar membrane, i.e.

$$g([CQ]_e - [CQ]_{DV}) = f([CQ]_{DV} - [CQ]_e) \quad \text{(eq-S2)}$$

where square brackets indicate concentrations and f(x) and g(x) are increasing functions of their arguments that are equal to zero when the argument is zero. The only possible solution of (eq-S2) is $[CQ]_{DV} = [CQ]_e$ (the analytical proof is conceptually identical to the one reported below in section S6). Notice that, in CQS strains, where PfCRT is not involved, the membrane equilibrium (expressed as: $g([CQ]_e - [CQ]_{DV}) = 0$) implies $[CQ]_{DV} = [CQ]_e$ as well. Therefore, the hypothesis that PfCRT is a channel for CQ entails that unprotonated chloroquine accumulation in the vacuole of CQR and CQS strains is the same. Having CQS and CQR strains identical pH_{DV} values [1] [2] [3], their vacuole must also have identical concentration of CQ^+ and CQ^{++}. As a consequence, the two strains also have the same value of $[CQ{:}HM]_{DV}$, regardless of the binding mechanism. In conclusion, the hypothesis that PfCRT is a channel for unprotonated CQ species implies that we should not observe any difference in CAR values between CQR and CQS strains, which is in contradiction with the experimentally observed CAR difference (cases B and C in Table 1, main text).

S2 - Membrane equilibrium equation

The CAR measurements reported by Bray *et al* [4] are taken after 10 minutes incubation of the cells in the presence of chloroquine. Given the known time evolution of chloroquine uptake [5], the system is expected to have reached a steady state. Consequently, the net chloroquine flux across the membrane of the infected erythrocyte is zero. The only form of chloroquine for which the erythrocyte and the plasmodium membranes are permeable is the un-protonated one and this implies that CQ concentrations are the same on the two sides of the membranes; this concentration is indicated here with $[CQ]_e$. In the case of CQ sensitive (CQS) strains this implies:

$[CQ]_e = [CQ]_{DV}$ (eq-S3)

On the other hand, for CQ resistant (CQR) strains, we must take into account the outward chloroquine flux across the vacuole membrane due to PfCRT (J_{PfCRT}). At steady state the inward diffusive flux of un-protonated chloroquine CQ is balanced by the outward flux due to PfCRT, i.e.

$$P_{cq}([CQ]_e - [CQ]_{DV}) = J_{PfCRT} \quad \text{(eq-S4)}$$

where P_{cq} is the permeability of the vacuolar membrane to CQ. Note that equation (eq-S4) becomes equation (eq-S3) when $J_{PfCRT} = 0$.

S3 - The expression for the Cellular Accumulation Ratio of chloroquine

We need an expression for the quantity measured in the experiments, i.e. for the Cellular Accumulation Ratio (CAR). Indicating the volume of the infected erythrocyte with V_{in}, the volume of the digestive vacuole with V_{DV} and the concentration of all the chloroquine inside the vacuole with $[C]_{DV}$, we have:

$$[C]_{DV} = [CQ]_{DV} + [CQ^+]_{DV} + [CQ^{++}]_{DV} + [CQ:HM]_{DV} \quad \text{(eq-S5)}$$

Defining $[C]_e = [CQ^{TOT}]_e$ as the concentration of chloroquine outside the vacuole:

$$[C]_e = [CQ^{TOT}]_e = [CQ]_e + [CQ^+]_e + [CQ^{++}]_e, \quad \text{(eq-S6)}$$

the average chloroquine concentration in the infected erythrocyte is

$$[C]_{in} = \frac{[C]_{DV} V_{DV} + [C]_e (V_{in} - V_{DV})}{V_{in}} \quad \text{(eq-S7)}$$

The total concentration of chloroquine in each compartment of the infected erythrocyte only depends on the pH of the compartment and on the concentration of the un-protonated chloroquine CQ. Recent measurements [1,2,3] have shown that the pH of the infected erythrocyte cytoplasm and of the plasmodium cytoplasm are very close to physiological pH. Thus, it is reasonable to assume that the pHs of the external medium, of the erythrocyte and of the plasmodium cytoplasm are equal. We indicate

this value as pH_e. Furthermore, due to the permeability of the membrane to CQ, at equilibrium the CQ concentration is equal in all compartments. The CQ concentration and pH being equal inside the erythrocyte and in the external buffer, we have $[C]_e = [C]_{out}$ (as more rigorously shown below in section S4). Therefore, the cellular accumulation ratio CAR can be expressed as:

$$CAR = \frac{[C]_{in}}{[C]_{out}} = \frac{[C]_{DV} V_{DV} + [C]_e (V_{in} - V_{DV})}{[C]_e V_{in}} \qquad \text{(eq-S8)}$$

S4 - Chloroquine concentration is equal in the external buffer and inside the erythrocyte

Let $[CQ^{TOT}]$ be the concentration of the total amount of free chloroquine, i.e.

$$[CQ^{TOT}] = [CQ] + [CQ^{+}] + [CQ^{++}] \qquad \text{(eq-S9)}$$

Combining (eq-S9) with eqs. (5) and (6) of the main text, we obtain

$$[CQ^{TOT}] = [CQ]\left(1 + \frac{[H^+]}{k'} + \frac{[H^+]^2}{k''}\right) \qquad \text{(eq-S10)}$$

which shows that the total amount of free chloroquine can be expressed as a function of both the pHs and the concentration of the un-protonated form, [CQ]. In our model we make the reasonable assumption that external buffer, erythrocyte cytoplasm, and plasmodium cytoplasm have the same pH. Moreover, at equilibrium, the three compartments have the same [CQ]. Hence, from eq (eq-S10), $[CQ^{TOT}]$ is the same both outside ($[C]_{out}$) and inside the infected erythrocyte ($[C]_e$), with the exception of the vacuole *lumen* where, due to the acidic pH and to the chloroquine-HM binding, the chloroquine total concentration ($[C]_{DV}$, see eq. (eq-S5)) is higher.

S5 - CAR expressions in the case of chloroquine-HM binding in linear regime (hypothesis H1)

CAR expressions corresponding to equations (8-10) of the main text are:

$$CAR = \frac{V_e + V_{DV}\frac{[CQ]_{DV}}{C_e}\left(1 + (1+\alpha)\frac{[H^+]_{DV}}{k'} + \frac{[H^+]_{DV}^2}{k''}\right)}{V_e + V_{DV}}$$

$$CAR = \frac{V_e + V_{DV}\frac{[CQ]_{DV}}{C_e}\left(1 + \frac{[H^+]_{DV}}{k'} + (\alpha+1)\frac{[H^+]_{DV}^2}{k''}\right)}{V_e + V_{DV}}$$

$$CAR = \frac{V_e + V_{DV}\frac{[CQ]_{DV}}{C_e}(1+\alpha)\left(1 + \frac{[H^+]_{DV}}{k'} + \frac{[H^+]_{DV}^2}{k''}\right)}{V_e + V_{DV}}$$

S6 - Proof that $[CQ]_{DV,D} = [CQ]_e$ in the hypothesis PfCRT is a channel for the protonated forms of chloroquine

Let J_{mem} be the inward membrane flux of unprotonated chloroquine CQ and J_{PfCRT} the chloroquine outward flux due to the CQR mutated form of PfCRT. At equilibrium eq (eq-S1) holds, where J_{mem} is a function of the chloroquine difference between the plasmodium cytoplasm and the vacuole, i.e. $J_{mem} = g([CQ]_e - [CQ]_{DV})$. In particular we have that J_{mem} has the following properties:

$J_{mem} = 0$ if $[CQ]_e = [CQ]_{DV}$ (eq-S11a)

$J_{mem} > 0$ if $[CQ]_e > [CQ]_{DV}$ (eq-S11b)

$J_{mem} < 0$ if $[CQ]_e < [CQ]_{DV}$ (eq-S11c)

Let us now consider the case in which J_{PfCRT} is a channel for CQ^+. In this case we have $J_{PfCRT} = f([CQ^+]_{DV} - [CQ^+]_e)$, and:

$J_{PfCRT} = 0$ if $[CQ^+]_{DV} = [CQ^+]_e$ (eq-S12a)

$J_{PfCRT} > 0$ if $[CQ^+]_{DV} > [CQ^+]_e$ (eq-S12b)

$J_{PfCRT} < 0$ if $[CQ^+]_{DV} < [CQ^+]_e$ (eq-S12c)

In experiment D, $pH_e = pH_{DV}$, hence

$$([CQ^+]_{DV} - [CQ^+]_e) = \frac{[H^+]([CQ]_{DV} - [CQ]_e)}{k'}$$

(eq-S12a), (eq-S12b) and (eq-S12c) imply

$J_{PfCRT} = 0$ if $[CQ]_{DV} = [CQ]_e$ (eq-S13a)

$J_{PfCRT} > 0$ if $[CQ]_{DV} > [CQ]_e$ (eq-S13b)

$J_{PfCRT} < 0$ if $[CQ]_{DV} < [CQ]_e$ (eq-S13c)

Combining (eq-S11a) with (eq-S13a), it is apparent that $[CQ]_{DV} = [CQ]_e$ is a solution of equation (eq-S1).

In the following we will show that this solution is unique, i.e. that no solution exists in the hypotheses $[CQ]_{DV} > [CQ]_e$ and $[CQ]_{DV} < [CQ]_e$.

We first consider $[CQ]_{DV} > [CQ]_e$. In this case, equation (eq-S11c) implies $J_{mem} < 0$ while equation (eq-S13b) implies $J_{PfCRT} > 0$, i.e. $J_{mem} \neq J_{PfCRT}$, which is not consistent with (eq-S1). Therefore, no solution of eq (eq-S1) exists if we assume $[CQ]_{DV} > [CQ]_e$. A similar argument holds in the hypothesis $[CQ]_{DV} < [CQ]_e$: Equation (eq-S11b) implies $J_{mem} > 0$ while equation (eq-S13c) implies $J_{PfCRT} < 0$, i.e. $J_{mem} \neq J_{PfCRT}$, that is not consistent with (eq-S1). Therefore no solution of eq (eq-S1) exists if we assume $[CQ]_{DV} < [CQ]_e$.

S7 - Value of the channel permeability in the hypothesis PfCRT is a channel and $[CQ:HM]_{DV} = \alpha[CQ]_{DV}$

The values of un-protonated chloroquine concentration and of PfCRT flux in experiment C have been derived in the main text and are $[CQ]_{DV,C} = 1.53 * 10^{-12}$ M and $J_{PfCRT} = 2.799 * 10^{-3}$ nM cm/sec, respectively. Using these values in the equation of outward PfCRT flux in the case in which PfCRT is a non saturated CQ^+ channel, i.e. $J_{PfCRT} = \beta([CQ^+]_{DV,C} - [CQ^+]_e)$, we obtain that the value of the channel permeability β is 2.7072 cm/s. A similar calculation gives $\beta = 4.816 * 10^{-3}$ cm/s in the case of PfCRT being a non saturated CQ^{++} channel, i.e. $J_{PfCRT} = \beta([CQ^{++}]_{DV,C} - [CQ^{++}]_e)$.

S8 - Calculation of CAR_D in the hypothesis $[CQ:HM]_{DV} = \alpha[CQ]_{DV}$ (H1) and

PfCRT is a non saturated active carrier (J2a) (main text, Figure 1, cells C3-5,5)

1) Hypothesis: $J_{PfCRT} = \lambda[CQ]_{DV}$ *,corresponding to cell* $C_{3,5}$ *(Figure 1, main text)*

The values of un-protonated chloroquine concentration and of PfCRT flux in experiment C have been derived in the main text and are $[CQ]_{DV,C} = 1.53 * 10^{-12}$ M and $J_{PfCRT} = 2.799 * 10^{-3}$ nM cm/sec, respectively. Using these values in the equation of the outward PfCRT flux

$$J_{PfCRT} = \lambda[CQ]_{DV,C} \qquad \text{(eq-S14)}$$

we obtain λ = 18.3cm/s. The CQR membrane balance in experiment D (obtained by combining eqs (eq-S4) and (eq-S14)) is:

$$P_{cq}\left([CQ]_e - [CQ]_{DV,D}\right) = \lambda[CQ]_{DV,D} \qquad \text{(eq-S15)}$$

and can be used to calculate $[CQ]_{DV,D}$. Substituting $[CQ]_{DV,D}$, λ and $pH_{DV} = pH_e$ in equation (11) of the main text, the CAR_D value can be easily obtained.

2) Hypothesis: $J_{PfCRT} = \lambda[CQ^+]_{DV}$*, corresponding to cell* $C_{4,5}$ *(Figure 1, main text)*

In this case the outward PfCRT flux is

$$J_{PfCRT} = \lambda\,[CQ^+]_{DV,C}$$

Using $[CQ]_{DV,C}$ and J_{PfCRT} values as derived in the main text, we obtain λ = 2.62 x 10^{-4} m/s. In this case, the CQR membrane balance in experiment D is:

$$P_{cq}\left([CQ]_e - [CQ]_{DV,D}\right) = \lambda[CQ^+]_{DV,D} = \frac{\lambda[H^+]_{DV}[CQ]_{DV,D}}{k'} \qquad \text{(eq-S16)}$$

where eq. (5) (main text) was used. Eq (eq-S16) can be used to calculate $[CQ]_{DV,D}$. Substituting $[CQ]_{DV,D}$, λ and $pH_{DV} = pH_e$ in equation (11) of the main text, the CAR_D value can be easily obtained.

3) Hypothesis: $J_{PfCRT} = \lambda[CQ^{++}]_{DV}$*, corresponding to cell* $C_{5,5}$ *(Figure 1, main text)*

The outward PfCRT flux is in this case

$$J_{PfCRT} = \lambda([CQ^{++}]_{DV,C})$$

Using $[CQ]_{DV,C}$ and J_{PfCRT} values as derived in the main text, we obtain $\lambda = 4.81 \times 10^{-7}$cm/s. In this case, the CQR membrane balance in experiment D, is:

$$P_{cq}\left([CQ]_e - [CQ]_{DV,D}\right) = \lambda[CQ^{++}]_{DV,D} = \frac{\lambda[H^+]^2_{DV}[CQ]_{DV,D}}{k"} \quad \text{(eq-S17)}$$

where eq. (6) (main text) was used. Eq (eq-S17) can be used to calculate $[CQ]_{DV,D}$. Substituting $[CQ]_{DV,D}$, λ and $pH_{DV} = pH_e$ in equation (11) of the main text of the main text, the CAR_D value is obtained.

S9 - Detailed numerical calculation showing that chloroquine-HM binding in experiment C is not in the saturation region

Let us consider the hypothesis that chloroquine-HM binding is in the saturation region in experiment A (cells $C_{1-5,1-4}$, Figure 1, main text). Combining equation (eq-S5) (eq-S6) and (eq-S8), for experiment A (where $pH_e = pH_{DV}$ and $[CQ]_e = [CQ]_{DV}$) the following expression for $[CQ{:}HM]_{DV,A}$ is obtained

$$[CQ:HM]_{DV,A} = C_e\left(CAR_A - 1\right)\frac{V_e + V_{DV}}{V_{DV}} \quad \text{(eq-S18)}$$

If we hypothesized that chloroquine-HM binding in experiment C is in the saturation region, we would have $[CQ{:}HM]_{DV,A} = [CQ{:}HM]_{DV,C}$ that, combined with (eq-S5) (eq-S6) (eq-S8) and (eq-S18), gives

$$CAR_C = CAR_A - 1 + \frac{C_eV_e + \left([CQ]_{DV} + [CQ^+]_{DV} + [CQ^{++}]_{DV}\right)V_{DV}}{C_e\left(V_{DV} + V_e\right)} \quad \text{(eq-S19)}$$

Since the third term of (eq-S19) is always positive, $CAR_C > CAR_A - 1$, which is not compatible with the experimental data.

S10 - Proof that eq. (18) (main text) is an increasing function of $[H^+]_{DV}$ in the interval $0 < pH_{DV} < 9.15$

In this section we demonstrate that eq. (18) of the main text is an increasing function of $[H^+]_{DV}$ in the interval $0 < pH_{DV} < 9.15$ for any value of λ and P_{cq}. For the sake of clarity we report here eq. (18)

$$[CQ^{TOT}]_{DV} = [CQ]_e \left(1 + \frac{\lambda [H^+]_{DV}}{P_{cq} k'}\right)^{-1} \left(1 + \frac{[H^+]_{DV}}{k'} + \frac{[H^+]_{DV}^2}{k''}\right)$$

The derivative of $[CQ^{TOT}]_{DV}$ with respect to $[H^+]_{DV}$ is

$$\frac{d[CQ^{TOT}]_{DV}}{d[H^+]_{DV}} = \frac{\left(\frac{1}{k'} + \frac{2[H^+]_{DV}}{k''}\right)\left(1 + \frac{\lambda [H^+]_{DV}}{P_{cq} k'}\right) - \frac{\lambda}{P_{cq} k'}\left(1 + \frac{[H^+]_{DV}}{k'} + \frac{[H^+]_{DV}^2}{k''}\right)}{\left(1 + \frac{\lambda [H^+]_{DV}}{P_{cq} k'}\right)^2} \qquad \text{(eq-S20)}$$

Being the denominator of (eq-S20) always positive, the sign of the derivative will be determined by the sign of its numerator that could be rewritten as

$$N = \frac{1}{k'} + \frac{2[H^+]_{DV}}{k''} + \frac{\lambda}{P_{cq} k'}\left(\frac{[H^+]_{DV}^2}{k''} - 1\right) \qquad \text{(eq-S21)}$$

The first two terms are always positive while the third term is positive if $[H^+]_{DV} > (k'')^{-1/2}$, i.e. $pH_{DV} < 9.15$. Therefore, for $pH_{DV} < 9.15$, the $[CQ^{TOT}]_{DV}$ expression given in eq. (18) of the main text is an increasing function of $[H^+]_{DV}$, regardless the value of λ and P_{cq}.

S11 – Calculation of CAR_D in saturated condition for chloroquine-HM binding and $J_{PfCRT} = \lambda[CQ]_{DV}$ (cells $C_{3,1\text{-}4}$, Figure 1 of the main text)

In this case the vacuolar membrane balance equation for CQR strains reads

P_{cq} ($[CQ]_e - [CQ]_{DV}$) = λ $[CQ]_{DV}$ (eq-S22)

Eq. (eq-S22) does not depend on the vacuolar pH_{DV} (which alone explains the difference between case C and case D) hence, being the extra-erythrocytic unprotonated chloroquine concentration ($[CQ]_e$) the same for all experiments, we have $[CQ]_{DV,D} = [CQ]_{DV,C}$. The same argument of section S14 below holds here, implying $CAR_D < CAR_C$, which is not compatible with experimental data. The corresponding cells are shaded in Figure 1 of the main text.

S12 - Calculation of CAR_D in saturated condition for chloroquine-HM binding and $J_{PfCRT} = \lambda[CQ^{++}]_{DV}$ (cells C5,1-4,Figure 1 of the main text])

In this case the vacuolar membrane balance equation for CQR parasites reads

P_{cq} $([CQ]_e - [CQ]_{DV}) = \lambda$ $[CQ^{++}]_{DV}$

that could be rewritten as

$$[CQ]_{DV} = [CQ]_e \left(1 + \frac{\lambda [H^+]^2_{DV}}{P_{cq} k''}\right)^{-1} \qquad \text{(eq-S23)}$$

Being $[CQ^{TOT}]$= [CQ] + $[CQ^+]$ + $[CQ^{++}]$ and using the dissociation relations (eqs (5) and (6), main text), we can write

$$[CQ^{TOT}]_{DV} = [CQ]_e \left(1 + \frac{\lambda [H^+]_{DV}}{P_{cq} k''}\right)^{-1} \left(1 + \frac{[H^+]_{DV}}{k'} + \frac{[H^+]^2_{DV}}{k''}\right)$$

This expression shows that the free chloroquine inside the vacuole is a function of pH_{DV} and of the ratio between the unknown parameter λ and the membrane permeability P_{cq}. Considering that $pH_{DV,C}$ and $pH_{DV,D}$ are known (in particular $pH_{DV,D}$ = $pH_{DV,e}$ while the $pH_{DV,C}$ value has been derived in the main text), the ratio $[CQ^{TOT}]_{DV,D}/[CQ^{TOT}]_{DV,C}$ is a function of λ / P_{cq} only. The dependence of $[CQ^{TOT}]_{DV,D}/[CQ^{TOT}]_{DV,C}$ from λ/P_{cq} is reported in figure S1 where it is evident that $[CQ^{TOT}]_{DV,D}/[CQ^{TOT}]_{DV,C}$ has an upper bound corresponding to its horizontal asymptote which is equal to 1.198. Hence we have

$$[CQ^{TOT}]_{DV,D} < 1.198*[CQ^{TOT}]_{DV,C} \qquad \text{(eq-S24)}$$

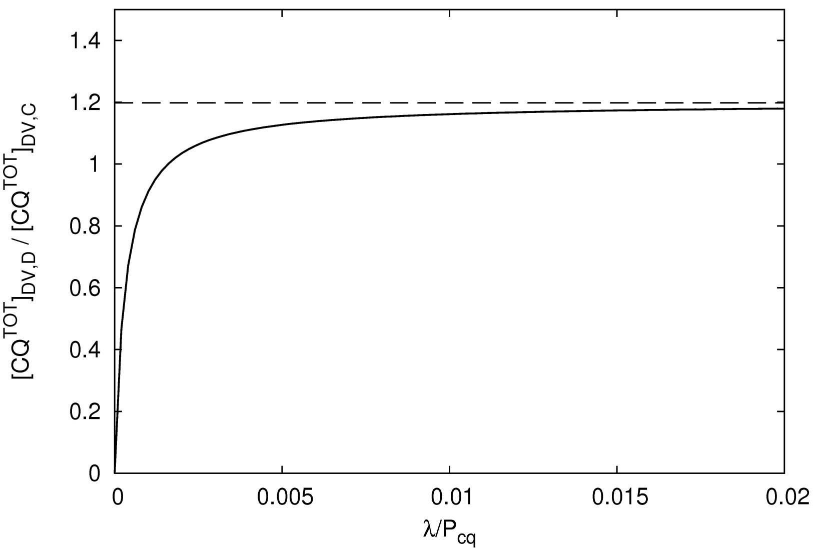

Figure S1 - Ratio $[CQ^{TOT}]_{DV,D}/[CQ^{TOT}]_{DV,C}$ as a function of λ/P_{cq}. The dashed line corresponds to the horizontal asymptote value $[CQ^{TOT}]_{DV,D}/[CQ^{TOT}]_{DV,C} = 1.98$

Let us now analyze separately each hypothesis for the CQ:HM binding:

1) $[CQ:HM]_{DV} = f([CQ]_{DV})$, corresponding to cell $C_{5,1}$ (Figure 1 of the main text)

Equation (eq-S23) shows that $[CQ]_{DV}$ is a decreasing function of $[H^+]$, hence, being $[H^+]_{DV,D} < [H^+]_{DV,C}$ we have $[CQ]_{DV,D} > [CQ]_{DV,C}$ and, consequently $[CQ:HM]_{DV,D} > [CQ:HM]_{DV,C}$. This expression, combined with (eq-S24) and eq (15) of the main text does not allow us to draw any conclusion on the relationship between CAR_D and CAR_C. In particular, (eq-S24) shows that $[CQ^{TOT}]_{DV,D} < 1.198*[CQ^{TOT}]_{DV,C}$ while we have demonstrated above that $[CQ:HM]_{DV,D} > [CQ:HM]_{DV,C}$. Hence cell $C_{5,1}$ cannot be shaded in Figure 1 of the main text.

2) $[CQ:HM]_{DV} = f([CQ^+]_{DV})$, corresponding to cell $C_{5,2}$ (Figure 1 of the main text)

Equation (eq-S23) combined with the equilibrium dissociation relation (eq (5), main text) can be rewritten as

$$[CQ^+]_{DV} = \left(\frac{[CQ]_e[H^+]_{DV}}{k'}\right)\left(1+\frac{\lambda[H^+]_{DV}^2}{P_{cq}k''}\right)^{-1} \qquad \text{(eq-S25)}$$

Equation (eq-S25), together with the known value of pH_{DV}, can be used to calculate the ratio $[CQ^+]_{DV,D}/[CQ^+]_{DV,C}$ as a function of λ/P_{cq} (Figure S2). This ratio is a bounded function ranging between the values 0.0091 and 109.64, which correspond to the value obtained when $\lambda/P_{cq} = 0$ and to the horizontal asymptote value, respectively. This implies that no conclusive prediction is possible for $[CQ:HM]_{DV,D}/[CQ:HM]_{DV,C}$ and, therefore, a conclusionon about the relationship between CAR_D and CAR_C cannot be drawn. This implies that the current hypothesis cannot be excluded and cell $C_{5,2}$ cannot be shaded in Figure 1 of the main text.

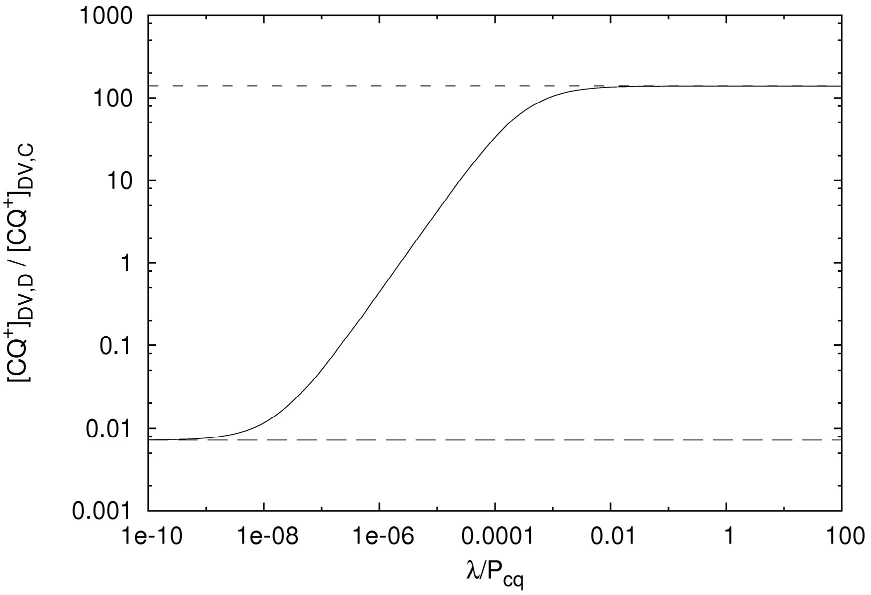

Figure S2 - Ratio $[CQ^+]_{DV,D}/[CQ^+]_{DV,C}$ as a function of λ/P_{cq}. The dashed lines correspond to the horizontal asymptote and to the value assumed for $\lambda/P_{cq} = 0$.

3) $[CQ:HM]_{DV} = f([CQ^{++}]_{DV})$, corresponding to cell $C_{5,3}$ (Figure 1 of the main text)

Equation (eq-S23) combined with equilibrium dissociation relation (eq (6), main text) can be rewritten as

$$[CQ^{++}]_{DV} = \left(\frac{[CQ]_e[H^+]^2_{DV}}{k''}\right)\left(1+\frac{\lambda[H^+]^2_{DV}}{P_{cq}k''}\right)^{-1} \qquad \text{(eq-S26)}$$

Eq (eq-S26) is an increasing function of $[H^+]$, which implies that $[CQ^{++}]_{DV,D} < [CQ^{++}]_{DV,C}$ and therefore $[CQ:HM]_{DV,D} < [CQ:HM]_{DV,C}$. Let us now demonstrate that

$CAR_D < 1.2\ CAR_C$. Equation (15) (main text) in case D reads

$$CAR_D = \frac{V_e}{V_e + V_{DV}} + \frac{V_{DV}[CQ^{TOT}]_{DV,D}}{C_e(V_e + V_{DV})} + \frac{V_{DV}[CQ:HM]_{DV,D}}{C_e(V_e + V_{DV})}$$

that, using eq (eq-S24) and $[CQ:HM]_{DV,D} < [CQ:HM]_{DV,C}$, gives

$$CAR_D < \frac{V_e}{V_e + V_{DV}} + 1.2\frac{V_{DV}[CQ^{TOT}]_{DV,C}}{C_e(V_e + V_{DV})} + \frac{V_{DV}[CQ:HM]_{DV,C}}{C_e(V_e + V_{DV})}$$

hence

$$CAR_D < 1.2\left(\frac{V_e}{V_e + V_{DV}} + \frac{V_{DV}[CQ^{TOT}]_{DV,C}}{C_e(V_e + V_{DV})} + \frac{V_{DV}[CQ:HM]_{DV,C}}{C_e(V_e + V_{DV})}\right) \qquad \text{(eq-S27)}$$

The expression in brackets on the right side of inequality (eq-S27) corresponds to CAR_C; therefore: $CAR_D < 1.2\ CAR_C$. Since this result is not compatible with the experimental data, we shaded the cell $C_{5,3}$ in Figure 1 of the main text.

4) $[CQ:HM]_{DV} = f([CQ^{TOT}]_{DV})$, corresponding to cell $C_{5,4}$ (Figure 1 of the main text)

Equation (eq-S24) implies $[CQ:HM]_{DV,D} < 1.198\ [CQ:HM]_{DV,C}$. Following a procedure similar to the one presented in the previous section, it is easy to demonstrate that, in this case, $CAR_D < 1.2\ CAR_C$. Again, being this result not compatible with the experimental data, we shaded cell $C_{5,4}$ in Figure 1 of the main text.

S13 - Calculations relating to each single hypothesis for the chloroquine:HM binding in saturated condition and $J_{PfCRT} = \lambda\ [CQ^+]_{DV}$ (cells C4,1-4, Figure 1 of the main text])

1) $[CQ:HM]_{DV} = f([CQ])$, corresponding to cell $C_{4,1}$ (Figure 1, main text)

Combining the membrane balance equation (eq (eq-S4)) and the expression for the PfCRT flux $J_{PfCRT} = \lambda([CQ^+]_{DV})$, we have:

$$[CQ]_{DV} = [CQ]_e \left(1 + \frac{\lambda [H^+]_{DV}}{P_{cq} k'}\right)^{-1} \qquad \text{(eq-S28)}$$

(eq-S28) is a decreasing function of $[H^+]_{DV}$. Hence, being $[H^+]_{DV,C} > [H^+]_{DV,D}$, it follows that $[CQ]_{DV,C} < [CQ]_{DV,D}$. The bound chloroquine concentration is an increasing function of [CQ], hence $[CQ{:}HM]_{DV,C} < [CQ{:}HM]_{DV,D}$. This result, combined with equation (15) of the main text and with the fact that equation (18) is an increasing function of $[H^+]_{DV}$ (see section S10), does not make it possible to predict whether CAR_C is larger or smaller that CAR_D. Accordingly, the corresponding cell in Figure 1 of the main text cannot be shaded.

2) $[CQ{:}HM]_{DV} = f([CQ^+])$, corresponding to cell $C_{4,2}$ (Figure 1, main text)

Using the chloroquine dissociation equilibrium (5) (main text), equation (eq-S28) can be rewritten as:

$$[CQ^+]_{DV} = \left(\frac{[CQ]_e [H^+]_{DV}}{k'}\right)\left(1 + \frac{\lambda [H^+]_{DV}}{P_{cq} k'}\right)^{-1}$$

that is an increasing function of $[H^+]_{DV}$. Hence, being $[H^+]_{DV,C} > [H^+]_{DV,D}$, it follows that $[CQ^+]_{DV,C} > [CQ^+]_{DV,D}$. The bound chloroquine concentration, is an increasing function of $[CQ^+]$ hence $[CQ{:}HM]_{DV,C} > [CQ{:}HM]_{DV,D}$. This result, combined with equation (15) of the main text and with the fact that equation (18) is an increasing function of $[H^+]_{DV}$ (see section S10) implies that CAR_C is larger than CAR_D, which is not consistent with experimental data. Accordingly, the corresponding cell in Figure 1 of the main text is shaded.

3) $[CQ{:}HM]_{DV} = f([CQ^{++}])$, corresponding to cell $C_{4,3}$ (Figure 1, main text)

Using the chloroquine dissociation equilibrium (6) (main text), equation (eq-S28) can be rewritten as:

$$[CQ^{++}]_{DV} = \left(\frac{[CQ]_e [H^+]_{DV}^2}{k''}\right)\left(1 + \frac{\lambda [H^+]_{DV}}{P_{cq} k'}\right)^{-1}$$

that is an increasing function of $[H^+]_{DV}$. Hence, being $[H^+]_{DV,C} > [H^+]_{DV,D}$, it follows that $[CQ^{++}]_{DV,C} > [CQ^{++}]_{DV,D}$. The bound chloroquine concentration is an increasing function of $[CQ^{++}]$, hence $[CQ{:}HM]_{DV,C} > [CQ{:}HM]_{DV,D}$. This result, combined with equation (15) of the main text and with the fact that equation (18) is an increasing function of $[H^+]_{DV}$ (see section S10) implies that CAR_C is larger than CAR_D, which is

not consistent with experimental data. Accordingly, the corresponding cell in Figure 1 of the main text is shaded.

4) $[CQ{:}HM]_{DV} = f([CQ^{TOT}])$, corresponding to cell $C_{4,4}$ (Figure 1, main text)

In section S10 we showed that $[CQ^{TOT}]$ is an increasing function of $[H^+]_{DV}$, hence, being $[H^+]_{DV,C} > [H^+]_{DV,D}$, it follows that $[CQ^{TOT}]_{DV,C} > [CQ^{TOT}]_{DV,D}$. The bound chloroquine concentration is an increasing function of $[CQ^{TOT}]$, hence $[CQ{:}HM]_{DV,C} > [CQ{:}HM]_{DV,D}$. This result, combined with equation (15) of the main text and with the fact that equation (18) is an increasing function of $[H^+]_{DV}$ (see section S10) implies that CAR_C is larger than CAR_D, which is not consistent with experimental data. Accordingly, the corresponding cell in Figure 1 of the main text is shaded.

S14 - Prediction for CAR_D in saturated condition for chloroquine-HM binding (H2) and J_{PfCRT} = const (J2b) (cells C2,1-4,Figure 1, main text)

Being J_{PfCRT} = const, we have that the vacuolar membrane balance equation for CQR strains reads

$$P_{cq}\,([CQ]_e - [CQ]_{DV}) = \text{const} \qquad \text{(eq-S29)}$$

The extra-erythrocytic unprotonated chloroquine concentration $[CQ]_e$ is the same for all the experiments hence, from eq. (eq-S29) we have $[CQ]_{DV,D} = [CQ]_{DV,C}$ where the second subscript indicates the experiment (A,B,C and D in Table 1 of the main text). The pH difference between experiments C and D, namely $pH_{DV,D} > pH_{DV,C}$, implies $[CQ^+]_{DV,D} < [CQ^+]_{DV,C}$ and $[CQ^{++}]_{DV,D} < [CQ^{++}]_{DV,C}$. Being the concentrations of the three free chloroquine forms obtained in case D lower or equal to those obtained in case C, we have $[CQ^{TOT}]_{DV,D} < [CQ^{TOT}]_{DV,C}$, where $[CQ^{TOT}] = [CQ] + [CQ^+] + [CQ^{++}]$. Moreover, $[CQ{:}HM]_{DV}$ in case D is equal (if $[CQ{:}HM]_{DV} = f([CQ])$) or lower (if $[CQ{:}HM]_{DV} = f([CQ^+])$ or $[CQ{:}HM]_{DV} = f([CQ^{++}])$) than the one obtained in case C. Hence, being both pH and HM-binding contributions, lower in case D than in case C, we have $CAR_D < CAR_C$ which is not compatible with experimental data. The corresponding cells are shaded in Figure 1 of the main text.

References

1. Hayward R, Saliba KJ, Kirk K (2006) The pH of the digestive vacuole of Plasmodium falciparum is not associated with chloroquine resistance. J Cell

Sci 119: 1016-1025.
2. Kuhn Y, Rohrbach P, Lanzer M (2007) Quantitative pH measurements in Plasmodium falciparum-infected erythrocytes using pHluorin. Cell Microbiol 9: 1004-1013.
3. Klonis N, Tan O, Jackson K, Goldberg D, Klemba M, et al. (2007) Evaluation of pH during cytostomal endocytosis and vacuolar catabolism of haemoglobin in Plasmodium falciparum. Biochem J 407: 343-354.
4. Bray PG, Mungthin M, Hastings IM, Biagini GA, Saidu DK, et al. (2006) PfCRT and the trans-vacuolar proton electrochemical gradient: regulating the access of chloroquine to ferriprotoporphyrin IX. Mol Microbiol 62: 238-251.
5. Sanchez CP, Stein W, Lanzer M (2003) Trans stimulation provides evidence for a drug efflux carrier as the mechanism of chloroquine resistance in Plasmodium falciparum. Biochemistry 42: 9383-9394.
