# Supplementary material for: On the Mechanism of Chloroquine Resistance in Plasmodium falciparum
Source: PLoS One. 2010 Nov 19;5(11):e14064. doi: 10.1371/journal.pone.0014064 (PMC2988812; doi:10.1371/journal.pone.0014064)
Supplement: Table S1 — HHpred results on Pfam and PDB databases. (0.02 MB PDF) [file pone.0014064.s002.pdf]

HHpred results on Pfam and PDB databases

target	probability	e-value	p-value	score	ss	% id
PF06027	100	1.20E-34	3.50E-39	213.8	26.8	18
PF08449	99.9	5.60E-24	1.60E-28	155.4	31.4	16
PF04142	99.9	7.80E-23	2.20E-27	149.1	23.3	12
PF03151	98.8	5.50E-09	1.60E-13	73.2	14.4	24
PF05653	98.3	4.30E-06	1.20E-10	57.3	17.1	11
PF00892	97.9	1.00E-05	2.90E-10	55.3	10.7	11
PF08449	96.7	0.0075	2.20E-07	39.5	14.4	9
PF06800	96.6	0.0037	1.10E-07	41.2	12.5	10
PF03151	96.6	0.0086	2.50E-07	39.2	14.2	12
3b5d_A	96	0.0085	2.40E-07	39.2	11.1	9
2i68_A	95.4	0.0037	1.10E-07	41.2	7.1	11
PF06027	93.8	0.14	3/E-6	32.6	15.3	8
PF00892	92.1	0.27	7.90E-06	31	11.5	13
PF08627	91	0.03	8.60E-07	36.3	4.5	15
PF06800	91	0.11	3.20E-06	33.1	7.6	12
PF04142	90.9	0.4	1.10E-05	30.1	11.5	14
PF00893	90.6	0.061	1.70E-06	34.6	5.8	9
PF06379	90.5	0.44	1.30E-05	29.8	12.3	11
PF04657	87	0.9	2.60E-05	28.1	15.4	18
PF05653	84.8	1.1	3.20E-05	27.6	9	11
PF10639	78	0.92	2.70E-05	28.1	6	9
2i68_A	71.9	4.1	0.00012	24.5	7.9	12
