# Supplementary material for: On the Mechanism of Chloroquine Resistance in Plasmodium falciparum
Source: PLoS One. 2010 Nov 19;5(11):e14064. doi: 10.1371/journal.pone.0014064 (PMC2988812; doi:10.1371/journal.pone.0014064)
Supplement: Table S2 — Phyre results. (0.02 MB PDF) [file pone.0014064.s003.pdf]

Phyre results

Template	E-value	Confidence
2CFQ	5.90E-07	100%
1PW4	2.00E-06	100%
2EXW	4.80E-06	95%
1OTS	9.10E-06	95%
1KPL	9.20E-06	95%
2GFP	1.90E-05	95%
