# Supplementary material for: On the Mechanism of Chloroquine Resistance in Plasmodium falciparum
Source: PLoS One. 2010 Nov 19;5(11):e14064. doi: 10.1371/journal.pone.0014064 (PMC2988812; doi:10.1371/journal.pone.0014064)
Supplement: Table S3 — PROCAIN results. (0.02 MB PDF) [file pone.0014064.s004.pdf]

PROCAIN results

template	score	e-value
1PW4	229	1.41E-05
2CFQ	196	7.29E-05
1HX6	167	1.66E-04
1MUK	222	2.72E-04
1TJV	168	5.39E-04
1DOF	157	6.70E-04
1RH5	152	6.99E-04
