# Supplementary material for: On the Mechanism of Chloroquine Resistance in Plasmodium falciparum
Source: PLoS One. 2010 Nov 19;5(11):e14064. doi: 10.1371/journal.pone.0014064 (PMC2988812; doi:10.1371/journal.pone.0014064)
Supplement: Table S4 — Shrimp results. (0.03 MB PDF) [file pone.0014064.s005.pdf]

shrimp results

Template	Score
1PW4_A	14.1275
2GFP_A	13.9516
2A65_A	13.0555
2A65_B	13.0555
2B2F_A	12.4628
2B2F_B	12.4628
2B2F_C	12.4628
1EHK_A	12.2762
1FFT_A	12.2705
1RH5_A	12.091
1ZCD_A	11.902
1AR1_A	11.3148
1U7G_A	11.2778
1U7G_B	11.2778
1U7G_C	11.2778
1M56_A	11.014
1V55_A	10.718
1V55_N	10.718
2FYN_A	10.7123
2FYN_D	10.7123
1QLE_A	10.2307
1BCC_C	9.5748
1BCC_M	9.5748
1KPL_B	9.1806
1L0L_C	8.9656
1L0L_N	8.9656
1OTS_A	8.4901
2NQ2_B	8.2244
1PP9_P	8.0748
2NQ2_A	8.0387

1PP9_C	8.0381
1OTS_B	7.8938
1KPL_A	7.4975
2IQV_A	7.1661
1XFH_A	6.8609
1XFH_B	6.8609
1XFH_C	6.8609
1V55_C	6.2207
1V55_P	6.2207
1M0L_A	6.1173
1M0L_B	6.1173
1M0L_C	6.1173
1PY6_A	6.0336
1M56_C	5.9471
2IQR_A	5.8651
1VGO_A	5.8518
2GIF_A	5.7555
2GIF_C	5.7555
2F2M_A	5.4891
2F2M_B	5.4891
2GIF_B	5.4111
1H68_A	5.218
2IC8_A	5.1981
1E12_A	5.1778
1E12_B	5.1778
1E12_C	5.1778
2IQL_A	4.9457
2IQO_A	4.9457
2NR9_A	4.5573
1ORQ_C	4.5545
1ORQ_D	4.5545
1ORQ_G	4.5545
1ORQ_J	4.5545

1H6I_A	4.3487
1H6I_B	4.3487
1H6I_C	4.3487
1H6I_D	4.3487
2A0L_A	4.1318
2A0L_B	4.1318
2A0L_G	4.1318
2A0L_H	4.1318
1LDF_A	4.09
1LDF_B	4.09
1LDF_C	4.09
1LDF_D	4.09
2B5F_A	3.8128
2B6P_A	3.688
2B6P_B	3.688
2B6P_C	3.688
2B6P_D	3.688
2D57_A	3.6306
2D57_B	3.6306
2D57_C	3.6306
2D57_D	3.6306
2B5F_D	3.5772
2B5F_C	3.5629
1WPG_A	3.5085
2B5F_B	3.5019
1Z98_A	3.404
1Z98_B	3.404
1Z98_C	3.404
1Z98_D	3.404
1VF5_A	3.3725
1VF5_N	3.3725
1SOR_A	2.7115
1SOR_B	2.7115

1SOR_C	2.7115
1SOR_F	2.7115
2B6O_A	2.7115
2B6O_B	2.7115
2B6O_C	2.7115
2B6O_F	2.7115
1FFT_C	2.7076
1Q90_C	2.6101
1Q90_I	2.6101
1YMG_A	2.4635
1YMG_B	2.4635
1YMG_C	2.4635
1YMG_D	2.4635
1PF4_A	2.3496
1PF4_B	2.3496
1Q16_C	1.4603
1Q16_F	1.4603
1YEW_B	0.9865
1YEW_F	0.9865
1YEW_J	0.9865
1KQF_C	0.9358
1KQF_F	0.9358
1KQF_I	0.9358
1YEW_C	0.8797
1YEW_G	0.8797
1YEW_K	0.8797
2A79_B	0.8028
2A79_F	0.8028
2A79_J	0.8028
2A79_N	0.8028
2HI7_B	0.605
2BBJ_A	0.5166
2BBJ_B	0.5166

2BBJ_D	0.5166
2BBJ_E	0.5166
2BBJ_F	0.5166
1NEK_D	0.3155
1NEK_E	0.3155
1NEK_J	0.3155
2AXT_A	0.1014
2AXT_A	0.1014
2BG9_E	-0.1689
2BG9_C	-0.1944
2AXT_D	-0.2089
2AXT_D	-0.2089
2BG9_B	-0.2172
2BG9_D	-0.3498
1MXM_A	-0.619
1MXM_B	-0.619
1MXM_C	-0.619
1MXM_D	-0.619
1MXM_E	-0.619
1MXM_F	-0.619
1MXM_G	-0.619
1Q90_K	-0.6949
1Q90_A	-0.73
1VF5_B	-1.2913
1VF5_O	-1.2913
1C17_M	-1.4942
1ZOY_C	-1.5588
1R3J_C	-1.9996
1R3J_D	-1.9996
1R3J_H	-1.9996
1R3J_J	-1.9996
1P7B_A	-2.1107
1P7B_B	-2.1107

1P7B_C	-2.1107
1P7B_D	-2.1107
1FFT_B	-2.2038
1S5H_C	-2.3301
1S5H_D	-2.3301
1S5H_H	-2.3301
1S5H_J	-2.3301
1M56_B	-2.4747
1MSL_A	-2.4821
1MSL_B	-2.4821
1MSL_C	-2.4821
1MSL_D	-2.4821
1MSL_E	-2.4821
1V55_B	-2.5009
1V55_O	-2.5009
1YQ3_C	-2.5757
1JB0_A	-2.7338
2AHY_B	-2.738
2AHY_C	-2.738
2AXT_B	-2.9219
2AXT_B	-2.9219
2HAC_A	-2.95
2HAC_B	-2.95
2AHY_D	-3.0004
1XL6_A	-3.0128
1XL6_B	-3.0128
1XL6_C	-3.0128
1XL6_D	-3.0128
2AXT_C	-3.8844
2AXT_C	-3.8844
1ORS_C	-3.9974
1A91_A	-4.0345
1ZOY_D	-4.1364

1H2S_B	-4.3017
1H2S_C	-4.3017
1YQ3_D	-4.4667
1IFP_B	-4.6267
1IFP_C	-4.6267
1IFP_D	-4.6267
1IFP_E	-4.6267
1IFP_F	-4.6267
1IFP_G	-4.6267
1IFP_H	-4.6267
1IFP_I	-4.6267
1IFP_J	-4.6267
1AFO_A	-4.6282
1AFO_B	-4.6282
2AXT_L	-4.8345
2AXT_L	-4.8345
1V55_L	-4.8612
1V55_Y	-4.8612
1JB0_L	-4.9898
1DXR_H	-5.1301
1Q90_B	-5.2654
1Q90_J	-5.2654
2AXT_E	-5.2995
2AXT_E	-5.2995
1IJD_C	-5.3402
1IJD_E	-5.3402
1IJD_I	-5.3402
1IJD_K	-5.3402
1IJD_O	-5.3402
1IJD_P	-5.3402
1EYS_H	-5.3723
1M56_D	-5.479
1IJD_A	-5.5057

1IJD_G	-5.5057
1IJD_M	-5.5057
1KB9_I	-5.5145
1KB9_S	-5.5145
1RZH_H	-5.5272
1IJD_B	-5.5714
1IJD_D	-5.5714
1IJD_F	-5.5714
1IJD_H	-5.5714
1IJD_J	-5.5714
1IJD_L	-5.5714
1IJD_N	-5.5714
1IJD_Q	-5.5714
1IJD_R	-5.5714
1NKZ_B	-5.5734
1NKZ_D	-5.5734
1NKZ_F	-5.5734
1NKZ_H	-5.5734
1NKZ_J	-5.5734
1NKZ_L	-5.5734
1NKZ_M	-5.5734
1NKZ_O	-5.5734
1NKZ_R	-5.5734
1QLE_D	-5.5739
1V55_I	-5.6638
1V55_V	-5.6638
2AXT_H	-5.667
2AXT_H	-5.667
1YEW_A	-5.7738
1YEW_E	-5.7738
1YEW_I	-5.7738
1RH5_C	-5.7955
1V55_D	-5.8157

1V55_Q	-5.8157
2AXT_K	-5.9015
2AXT_K	-5.9015
1KB9_H	-5.9481
1KB9_L	-5.9481
1LGH_B	-5.9687
1LGH_E	-5.9687
1LGH_F	-5.9687
1LGH_H	-5.9687
1LGH_J	-5.9687
1LGH_L	-5.9687
1LGH_N	-5.9687
1LGH_P	-5.9687
1KB9_D	-5.9852
1KB9_Q	-5.9852
1V55_K	-5.9858
1V55_X	-5.9858
2AXT_F	-5.9974
2AXT_F	-5.9974
1EHK_B	-5.9982
1ZZA_A	-6.0012
1RH5_B	-6.049
2FYN_C	-6.1532
2FYN_F	-6.1532
1NKZ_A	-6.188
1NKZ_C	-6.188
1NKZ_E	-6.188
1NKZ_G	-6.188
1NKZ_I	-6.188
1NKZ_K	-6.188
1NKZ_N	-6.188
1NKZ_P	-6.188
1NKZ_Q	-6.188

1PP9_G	-6.2634
1VF5_D	-6.3403
1VF5_Q	-6.3403
1PP9_T	-6.3623
1Q90_D	-6.4006
1Q90_L	-6.4006
1P49_A	-6.4198
1KQF_B	-6.6245
1KQF_D	-6.6245
1KQF_H	-6.6245
2J58_A	-6.701
2J58_B	-6.701
2J58_C	-6.701
2J58_D	-6.701
2J58_E	-6.701
2J58_F	-6.701
2J58_G	-6.701
2J58_H	-6.701
1Q90_F	-6.8008
1Q90_N	-6.8008
1BCC_J	-6.8346
1BCC_T	-6.8346
1L0L_J	-6.8358
1L0L_U	-6.8358
1KB9_E	-6.8537
1KB9_O	-6.8537
1JB0_K	-7.0129
2J7A_I	-7.0772
2J7A_L	-7.0772
1BCC_Q	-7.0963
1BCC_G	-7.0989
1OKC_A	-7.1162
1YM6_A	-7.1162

2BHW_A	-7.1919
2BHW_B	-7.1919
2BHW_C	-7.1919
1RWT_A	-7.2002
1RWT_B	-7.2002
1RWT_C	-7.2002
